# Partial downregulation of platelet glycoprotein VI by low affinity antibodies confers sustained and safe antithrombotic protection

**DOI:** 10.1038/s41392-026-02864-5

**Published:** 2026-07-01

**Authors:** Stefano Navarro, Sarah Beck, Sabrina I. Bonfiglio, Ernesto J. Cuenca-Zamora, Lukas J. Weiß, Marijke Kuijpers, Johan Heemskerk, David Stegner, Bernhard Nieswandt

**Affiliations:** 1 Institute of Experimental Biomedicine I, University Hospital Würzburg, Würzburg, Germany; 2 Rudolf Virchow Center, Center for Integrative and Translational Bioimaging, Julius-Maximilians University Würzburg Josef-Schneider-Str. 2, Würzburg, Germany; 3 Department of Internal Medicine I, University Hospital Würzburg, Würzburg, Germany; 4 Department of Biochemistry, Cardiovascular Research Institute Maastricht (CARIM), Maastricht University, Maastricht, The Netherlands; 5 Synapse Research Institute, Maastricht, The Netherlands; 6 Emfret Analytics GmbH & Co. KG, Eibelstadt, Germany

**Keywords:** Drug regulation, Biologics

## Abstract

Glycoprotein (GP) VI is a platelet-specific activating receptor for collagen and fibrin(ogen), and a promising target for antithrombotic therapy. Inhibitory Fab fragments against GPVI provide robust protection in mouse models of arterial thrombosis and ischemic stroke without impairing hemostasis in mice or humans. However, their short in vivo half-life limits their suitability for long-term therapy. In contrast, GPVI-targeting IgGs (e.g., JAQ1-3) induce complete receptor depletion and a GPVI-knockout-like (GPVI^KO-like^) phenotype in mice, a mechanism also observed in humans with anti-GPVI autoantibodies. While GPVI deficiency causes only moderate hemostatic defects, bleeding risk increases when combined with high-dose aspirin (100 mg/kg). Using a humanized GPVI (*hGP6*^*tg/tg*^) mouse model, we show that high-affinity anti-human GPVI IgGs (Emf1 K_D_ 0.490 nM, Emf2) induce a GPVI^KO-like^ phenotype with antithrombotic efficacy. In contrast, JAQ1 IgG, which binds hGPVI with low affinity (K_D_: 9.6 nM), induces only partial (~50%) GPVI downregulation, generating a stable low-density GPVI (GPVI^LO^) phenotype lasting up to 10 days. GPVI^LO^ platelets showed impaired aggregation, abolished procoagulant activity, and—similar to Fab-mediated inhibition—conferred protection from arterial thrombosis and LPS-induced thrombo-inflammation without increasing bleeding, even when combined with high-dose aspirin. The low-affinity anti-mouse GPVI antibody JAQ4 (K_D_ = 21 nM) induced a comparable GPVI^LO^ phenotype in wild-type mice. Transfusion of human platelets in NOD/SCID mice showed that complete and partial GPVI depletion, respectively, also occurs in human platelets in vivo. These findings establish that partial, affinity-dependent GPVI downregulation can provide sustained antithrombotic protection while preserving hemostasis, offering a promising strategy for long-term platelet inhibition.

## Introduction

Platelet activation and aggregation are essential for normal hemostasis, but also a major pathomechanism underlying life-threatening ischemic disease states such as myocardial infarction (MI) or acute ischemic stroke (AIS) which remain the leading causes of death and severe disability worldwide.^1,2^ Therefore, antiplatelet drugs have become indispensable therapeutics to efficiently prevent or treat arterial thrombosis, but they all bear an inherent risk of bleeding, especially in patients with comorbidities requiring dual platelet inhibition or concomitant anticoagulation. Thus, the development of new anti-thrombotic treatment regimens exhibiting a reduced risk of bleeding without losing drug efficacy is highly demanded.^3,4^

Among platelet receptors, GPVI has emerged as a promising pharmacological target, as its absence or functional inhibition is highly protective in models of arterial thrombosis, but also thrombo-inflammatory pathologies such as AIS^5–7^ or acute lung injury (ALI)^8^ in different mammalian model organisms in the absence of major bleeding complications.^6,9,10^ GPVI is a ~65 kDa platelet and megakaryocyte-specific collagen/fibrin(ogen) receptor that non-covalently associates with the immunoreceptor tyrosine-based activation motif (ITAM)-containing Fc receptor γ-subunit (FcRγ-chain) in the plasma membrane.^11^ Signaling is induced by receptor clustering on multivalent ligands and results in powerful platelet activation characterized by shape change, upregulation of integrin function, granule release and surface exposure of procoagulant phosphatidylserine (PS). Strength of GPVI signaling is greatly dependent on receptor density in the platelet membrane^12^ and high GPVI surface expression levels are associated with an increased risk of thrombosis, stroke, and acute coronary syndrome.^13–15^

The first antibody-based GPVI inhibitors have been developed and (pre-)clinically tested, most notably the humanized antibody Fab fragments EMA601^16^ and ACT017 (Glenzocimab)^17^ which appear to inhibit GPVI function through a similar mechanism, although with different potency.^16,18^ Both Fabs interfere with GPVI signaling while leaving ligand binding intact, thus allowing platelet adhesion while blocking or reducing, respectively, cellular activation through the receptor.^16,19^ In experimental animals, EMA601 and Glenzocimab did not affect bleeding times or induce any signs of increased bleeding tendency.^16,20^ This was also confirmed in first clinical studies with Glenzocimab, showing that the inhibitor was safe in healthy volunteers,^17^ but also in patients with AIS.^21^ Due to rapid renal clearing, Fabs like EMA601 and Glenzocimab have a relatively short half-life in vivo, which may be desirable in the treatment of acute conditions, but not suitable for long-term GPVI inhibition.

We have previously shown that the treatment of mice with anti-GPVI antibodies (JAQ1, 2, 3) results in immunodepletion of GPVI from circulating platelets and a GPVI knockout-like (GPVI^KO-like^) phenotype that persists for up to two weeks.^10,22–24^ Similarly, infusion of human platelets opsonized with an anti-hGPVI antibody in mice also led to GPVI downregulation.^23^ This immunodepletion is Fc-dependent and occurs in the liver through a mechanism involving the inhibitory Fcγ receptor (FcγR)IIB expressed on liver sinusoidal endothelial cells (LSECs).^22^ Similar to *Gp6*^*-/-*^ mice, such GPVI^KO-like^ mice are strongly protected in models of thrombosis and thrombo-inflammation while bleeding times are only moderately increased. However, when GPVI depletion was combined with high-dose aspirin (ASA, 100 mg/kg) severely increased bleeding times were observed.^25^ GPVI immunodepletion has also been reported in patients who had developed anti-GPVI autoantibodies,^26–30^ resulting in a sustained GPVI deficiency and mild to moderate bleeding defects similar to those reported in humans with a genetic GPVI deficiency.^31^ Thus, in contrast to Fab-mediated GPVI inhibition the complete loss of GPVI from circulating platelets has a significant impact on normal hemostasis that may be disadvantageous for long-term treatment in humans.^26–29,32^

We here show that low affinity anti-GPVI antibodies consistently induce partial GPVI depletion in *hGP6*^*tg/tg*^ as well as in wild-type mice, thereby maintaining GPVI^LO^ platelets in the circulation. Mice with GPVI^LO^ platelets were protected from arterial thrombosis as well as LPS-induced pulmonary thrombo-inflammation but did not exert the bleeding defect seen in GPVI^KO-like^ mice. Notably, transfusion of human platelets in NOD/SCID mice demonstrated that the differential downregulation is similarly inducible in human platelets in vivo.

## Results

### Emf1 induces complete hGPVI immunodepletion in *hGP6*^*tg/tg*^ mice

**Fig. 1:**
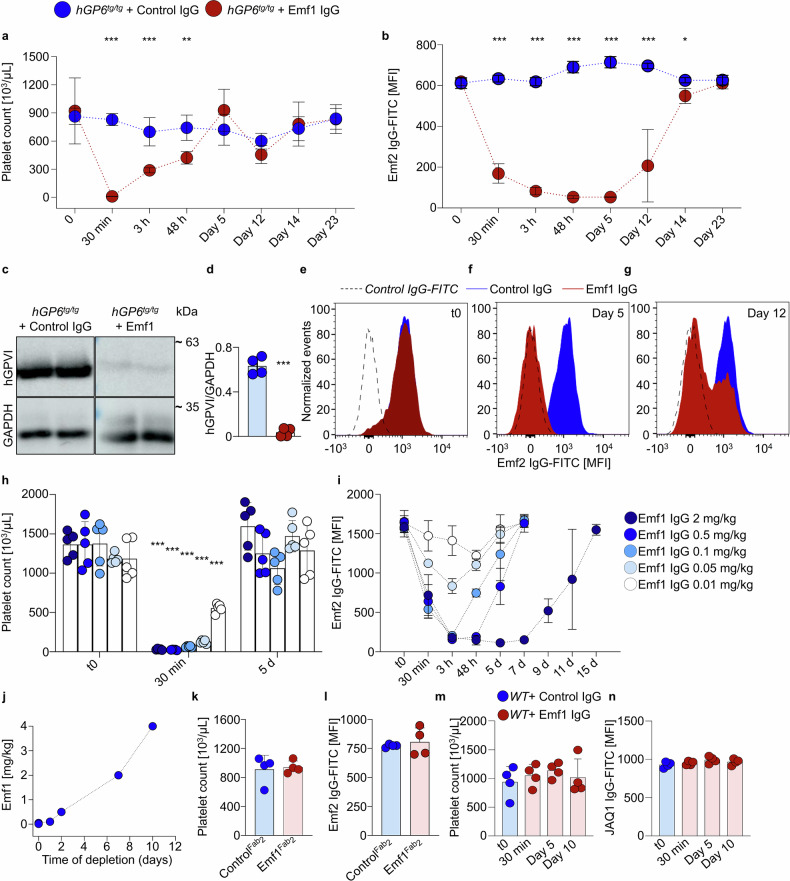
Emf1 IgG induces a hGPVI^KO-like^ phenotype in *hGP6*^*tg/tg*^ mice. **a**, **b** *hGP6*^*tg/tg*^ animals (*n* = 5) received 4 mg/kg Emf1 or control IgG. Platelet counts (**a**) were measured using an automated cell counter; surface GPVI levels were determined by flow cytometry using Emf2-FITC (**b**). **c**, **d** Western blot analysis of hGPVI expression detected with Emf2 in platelet lysates with GAPDH as loading control at 5 d post-treatment. Representative blots (**c**) and densitometric quantification (**d**) are shown. **e**–**g** Flow cytometric profiling of GPVI abundance on platelets ex vivo at baseline (**e**), day 5 (**f**), or day 12 (**g**) post-injection; dashed lines indicate isotype controls. **h**–**j** Dose–response assessment of Emf1: platelet count (**h**), GPVI expression (**i**), and correlation of dose and duration of the hGPVI^KO^ phenotype (**j**). **k**, **l** Effect of 4 mg/kg Emf1^Fab2^ on platelet count (**k**) and GPVI surface levels (**l**) 1 h post-treatment. **m**, **n** *WT* mice treated with 4 mg/kg Emf1 showed no reduction in platelet count (**m**) or GPVI expression (**n**), confirming antibody specificity for hGPVI. Statistical analyses: Two-way ANOVA with Bonferroni correction (**a**, **b**, **h**), Mann–Whitney U test (**d**); data are expressed as mean ± SD; *p < 0.05, **p < 0.01, ***p < 0.001

To assess whether hGPVI can be fully downregulated in vivo, we treated *hGP6*^*tg/tg*^ mice with the anti-hGPVI mAb Emf1^33^ (4 mg/kg i.v.) which resulted in a transient drop of platelet counts (Fig. 1a). Flow cytometric analysis of circulating platelets with Emf2^FITC^, which binds to a different epitope on hGPVI than Emf1,^34^ showed a complete absence of hGPVI from the plasma membrane already 30 min after treatment that lasted up to day 12. This was followed by a progressive increase in hGPVI levels and full recovery on day 23 (Fig. 1b). Western blot analysis confirmed the virtually complete absence of hGPVI in Emf1-treated *hGP6*^*tg/tg*^ mice on day 5 after injection (Fig. 1c, d), which was further confirmed by flow-cytometry, demonstrating the generation of a GPVI^KO-like^ phenotype (Fig. 1e, f). With the recovery of GPVI expression on day 12 post-injection, flow cytometry revealed two platelet populations: one lacking surface hGPVI and one with levels comparable to controls (Fig. 1g). Notably, Emf1 induced complete depletion of hGPVI even at doses as low as 0.1 mg/kg, with progressively shorter absence of GPVI observed at lower doses, indicating a direct correlation between antibody dose and duration of GPVI deficiency (Fig. 1h–j). We further confirmed that Emf1 induces the hGPVI^KO-like^ phenotype via a Fc-dependent mechanism, as its F(ab)_2_ fragment caused neither transient thrombocytopenia nor receptor depletion (Fig. 1k, l). To assess cross-reactivity with mGPVI, we administered Emf1 to *WT* mice, but observed no changes in platelet count or GPVI surface expression, confirming that the antibody targets human, but not mouse GPVI (Fig. 1m, n).

Previous studies demonstrated that the immunodepletion of mGPVI in *WT* mice occurs independently of the exact binding epitope of the anti-GPVI antibody.^24^ To test whether this epitope-independence also applies to hGPVI, we treated *hGP6*^*tg/tg*^ mice with Emf2.^34^ Flow cytometric cross-inhibition analysis confirmed that Emf1 and Emf2 bind to non-overlapping epitopes (Supplementary Fig. 1a, b). Emf2 did not induce thrombocytopenia or GPVI downregulation in *WT* mice, confirming its specificity for hGPVI (Supplementary Fig. 1c, d). As expected, Emf2-treated *hGP6*^*tg/tg*^ mice underwent transient thrombocytopenia and downregulation of the receptor with similar kinetics and efficacy as seen with Emf1, although with slightly faster recovery of surface hGPVI abundance (Supplementary Fig. 1e, f). Finally, Emf2^Fab2^ did not affect platelet count or receptor abundance in *hGP6*^*tg/tg*^ mice (Supplementary Fig. 1g, h).

**Fig. 2:**
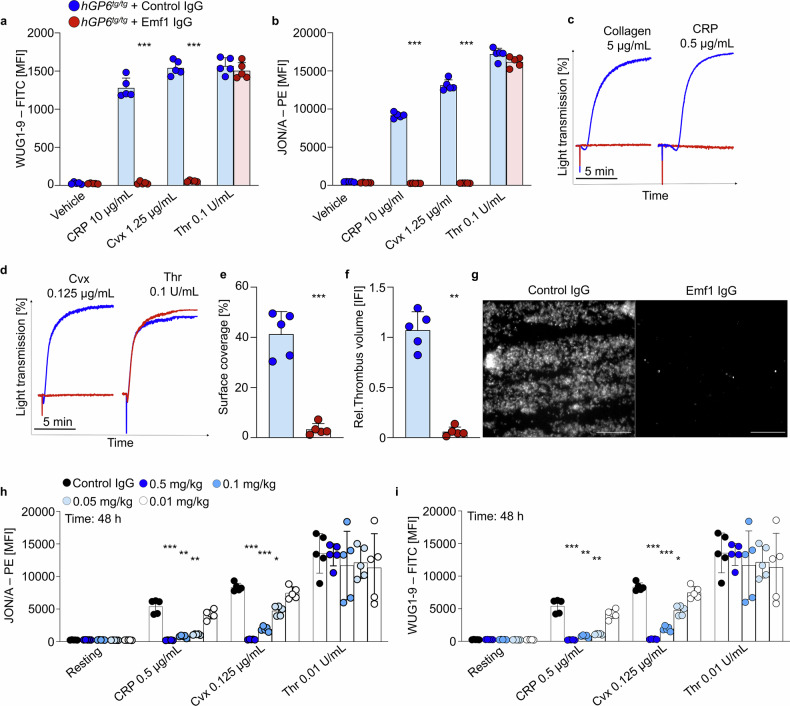
hGPVI^KO-like^ platelets are functionally unresponsive to GPVI-specific agonists. *hGP6*^*tg/tg*^ mice treated with 4 mg/kg Emf1 or control IgG were analyzed on day 5. **a**, **b** Activation of platelets determined by flow cytometry by measurement of P-selectin expression (**a**) and activated integrin αIIbβ3 (JON/A^PE^) (**b**) following stimulation with indicated agonists. **c**, **d** Aggregometry responses of washed platelets to the indicated agonists (*n* = 6); Cvx = convulxin, Thr = thrombin. **e**–**g** Platelet adhesion (**e**) and aggregate formation (**f**) on Horm collagen under flow (1000 s^−1^) in heparinized blood from young (8 weeks, 3 male and 2 female) *hGP6*^*tg/tg*^ mice; **g** Representative images are shown; scale bar: 50 µm. **h**, **i** *hGP6*^*tg/tg*^ animals were treated with the indicated dose of Emf1 and platelet function was assessed ex vivo at 48 h after injection. Activation of platelet αIIbβ3 integrin (JON/A^PE^) (**h**) and degranulation (α-P-selectin^FITC^) (**i**) was determined by flow cytometry upon stimulation with the indicated agonists. Data were analyzed using the two-way Anova (**a**, **b**, **h**, **i**) or the Mann-Whitney U Test (**e**, **f**). Data are expressed as mean ± SD; significance is expressed as *p < 0.05, **p < 0.01, ***p < 0.001, vs. indicated group

In agreement with data previously reported for mGPVI-depleted platelets,^10^ hGPVI^KO-like^ platelets were unresponsive to all tested concentrations of collagen-related peptide (CRP) as well as convulxin (CVX) as measured by P-selectin exposure and αIIbβ3 integrin activation (JON/A^PE^ binding). This was confirmed by an abolished aggregation response to CRP, CVX and collagen, while in all cases response to thrombin was unaltered (Fig. 2a–d and Supplementary Fig. 2a–e). Furthermore, aggregate formation of these platelets on collagen under arterial shear rate (1000s^−1^) was abolished (Fig. 2e–g). Notably, lower doses of Emf1 (down to 0.5 mg/kg) were also sufficient to induce a stable hGPVI^KO-like^ phenotype with complete blockade of GPVI-dependent platelet activation (Fig. 2h, i). These results showed that hGPVI can be efficiently downregulated in circulating platelets in vivo thereby inducing a GPVI^KO-like^ phenotype as previously reported for the treatment of *WT* mice with JAQ1, 2 or 3.^10^

### JAQ1 binds hGPVI with low affinity and induces partial hGPVI depletion in vivo

**Fig. 3:**
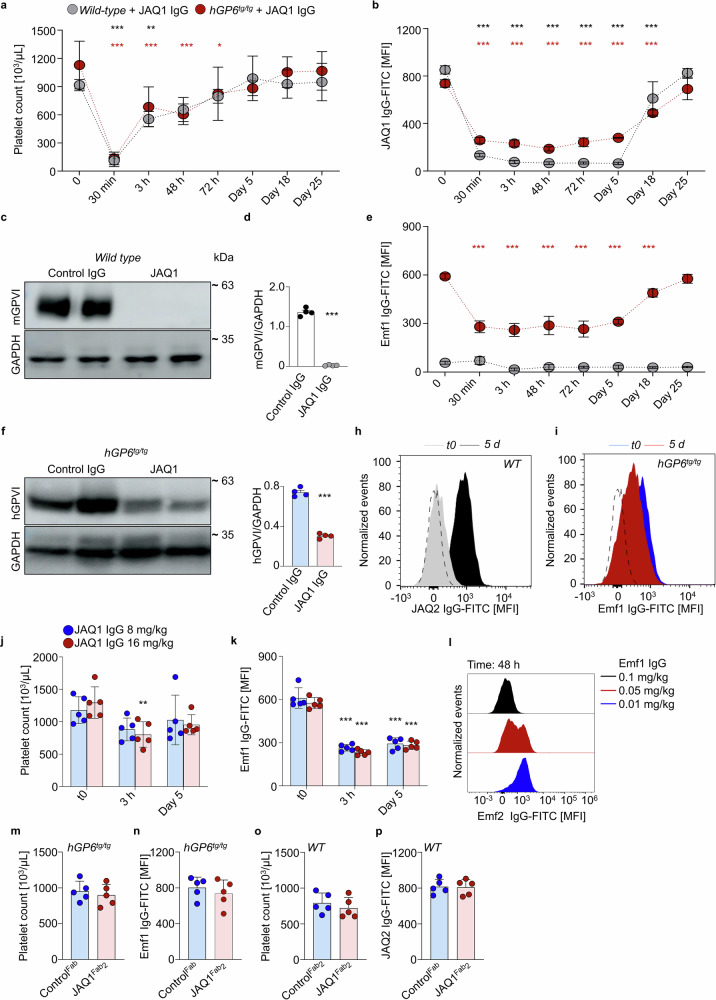
JAQ1 IgG induces a stable low-density GPVI phenotype (hGPVI^LO^). **a**, **b** *WT* or *hGP6*^*tg/tg*^ animals (*n* = 5) treated with 4 mg/kg b.w. JAQ1. Platelet count (**a**) was determined with an automatic cell counter while GPVI expression (**b**) was determined by flow cytometry. **c**, **d** Western blot of mGPVI in *WT* platelets detected using JAQ2; representative bands (**c**) and quantification (**d**) are shown. **e**–**g** hGPVI levels measured by flow-cytometry using Emf1^FITC^ (**e**) and Western blot using Emf1 (**f**, **g**) in *hGP6*^*tg/tg*^ mice at 5 d post JAQ1 or control IgG. **h**, **i** Flow cytometric receptor profiling in *WT* (**h**) and *hGP6*^*tg/tg*^ (**i**) mice pre- and post-treatment. **j**, **k** Dose escalation of JAQ1 (8 and 16 mg/kg): platelet count (**j**) and GPVI levels (**k**). **l** GPVI abundance measured by flow cytometry after Emf1 dose–response experiment in *hGP6*^*tg/tg*^ animals (48 h post-injection). **m**–**p** JAQ1-F(ab)_2_ (4 mg/kg) in *hGP6*^*tg/tg*^ (**m**, **n**) or *WT* (**o**, **p**) mice 1 h after injection: platelet count (**m**–**o**), GPVI expression (**n**–**p**). Statistics: Two-way ANOVA followed by Bonferroni’s multiple comparison test (**a**, **b**, **j**, **k**) and Mann-Whitney U Test (**d**, **g, m**–**p**). Data are expressed as mean ± SD; significance is expressed as *p < 0.05, **p < 0.01, ***p < 0.001, vs. indicated group

We have previously shown that the anti-mGPVI mAb, JAQ1, cross-reacts with hGPVI but does not inhibit its function.^34^ To test whether this discrepancy also results in differential receptor downregulation, we injected *WT* and *hGP6*^*tg/tg*^ mice with 4 mg/kg JAQ1 IgG i.v. and monitored platelet count and function as well as GPVI expression at different time intervals thereafter.^34^ In agreement with previous reports,^10^ all mice underwent a transient thrombocytopenia after 30 min followed by a rapid reversal and a full recovery on day 5 (Fig. 3a). Detection with JAQ1^FITC^ by flow cytometry and Western blot analysis showed complete depletion of GPVI in *WT* mice, but only partial downregulation of the human receptor in *hGP6*^*tg/tg*^ animals (Fig. 3b–d). Flow cytometric detection of Emf1^FITC^ binding revealed that hGPVI levels had decreased to ~47.4% of control where they remained for up to 5 days, which was also confirmed by Western blot analysis (Fig. 3e–g). The flow cytometry data also clearly showed that the JAQ1 treatment of *hGP6*^*tg/tg*^ mice had generated a homogeneous population of low-density hGPVI (hGPVI^LO^) platelets, rather than two populations (Fig. 3h, i). Interestingly, increasing the JAQ1 dose to 8 or 16 mg/kg did not further reduce hGPVI levels, suggesting a saturable threshold for GPVI downregulation by JAQ1 (Fig. 3j, k). In contrast, very low doses of Emf1 (0.05 mg/kg) failed to induce a stable GPVI^LO^ phenotype, instead causing complete depletion in only a subset of platelets, leading to a short-lived intermediate state (Figs. 1h, i and 3l). Finally, JAQ1-F(ab)_2_ failed to downregulate GPVI in either *WT* or *hGP6*^*tg/tg*^ mice, consistent with previous findings for Emf1 and Emf2, and confirming that both complete (GPVI^KO-like^) and partial (GPVI^LO^) depletion occur through a Fc-dependent mechanism (Fig. 3m–p). Together, these data demonstrated that JAQ1 consistently induces partial immunodepletion of hGPVI in vivo, resulting in a GPVI^LO^ phenotype of circulating platelets.

### Markedly reduced responses of hGPVI^LO^ platelets to GPVI-specific agonists

**Fig. 4:**
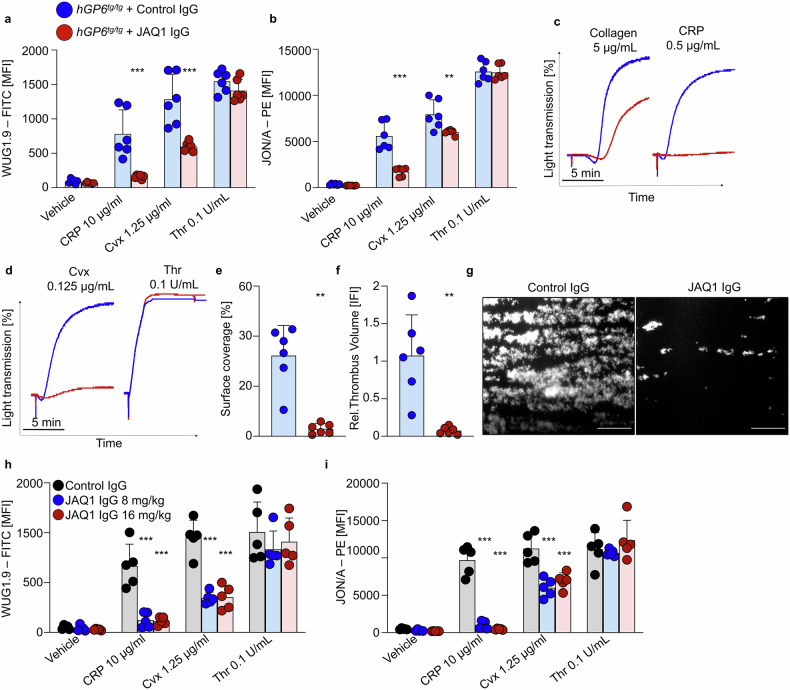
hGPVI^LO^ platelets exhibit attenuated GPVI signaling responses. *hGP6*^*tg/tg*^ mice treated with 4 mg/kg JAQ1 or control IgG were analyzed on day 5. **a**, **b** Flow cytometry of P-selectin exposure (**a**) and αIIbβ3 activation (**b**) upon stimulation. **c**, **d** Aggregation responses of washed platelets (*n* = 6); Cvx = convulxin; Thr = thrombin. **e**–**g** Platelet adhesion (**e**) and aggregate formation (**f**) under flow (1000 s^−1^) on Horm collagen (200 µg/mL) in heparinized blood from young (8 weeks) *hGP6*^*tg/tg*^ mice (3 male and 3 female mice); (**g**) Representative images are shown; scale bar: 50 µm. **h**, **i** Dose–response analysis 48 h post JAQ1 injection: flow cytometric detection of P-selectin exposure (**h**) and αIIbβ3 activation (**i**). Statistics: Kruskal–Wallis test (**a**, **b**, **h**, **i**) or the Mann-Whitney U Test (**e**, **f**). Data are expressed as mean ± SD; significance is expressed as **p < 0.01, ***p < 0.001, vs. indicated group

To assess the function of hGPVI^LO^ platelets, we injected *hGP6*^*tg/tg*^ mice with 4 mg/kg JAQ1 or control IgG and analyzed the platelets ex vivo on day 5. Flow cytometric analysis showed that hGPVI^LO^ platelets were virtually unresponsive to all tested concentrations of CRP, while their activation response to the more potent GPVI agonist CVX was significantly impaired. In contrast, thrombin-induced activation remained unaffected (Fig. 4a, b and Supplementary Fig. 2a, b). In standard light transmission aggregometry, hGPVI^LO^ platelets displayed strongly reduced responses to CRP (1, 0.5, and 0.1 µg/mL) and CVX (0.250, 0.125, and 0.075 µg/mL). Aggregation was significantly reduced at high collagen concentrations (10 and 5 µg/mL) and abolished at low concentrations (2.5 µg/mL). Again, thrombin-induced aggregation remained unaffected, confirming the selective effect on GPVI agonists (Fig. 4c, d and Supplementary Fig. 2c–e). In a whole blood perfusion assay on collagen (200 µg/mL) under arterial shear rates (1000 s^−1^) hGPVI^LO^ platelets from young mice displayed impaired aggregate formation indicative of impaired GPVI activity (Fig. 4e–g). Notably, platelets from *hGP6*^*tg/tg*^ mice treated with 8 or 16 mg/kg JAQ1 exhibited activation profiles similar to platelets from the 4 mg/kg JAQ1-treated group, thus revealing no further functional suppression despite higher antibody doses (Fig. 4h, i).

Together, these results demonstrated that the partial depletion of hGPVI in platelets is sufficient to markedly reduce GPVI-dependent platelet activation and thrombus formation under flow confirming that GPVI expression levels greatly determine platelet responsiveness to collagen.^34^

### Partial hGPVI depletion occurs independently of receptor density

To assess whether the only partial depletion of hGPVI was, at least in part, determined by hGPVI density in the platelet membrane, we studied mice heterozygous for hGPVI (*hGP6*^*wt/tg*^), which express 50% mGPVI and 50% hGPVI. *hGP6*^*wt/tg*^ mice treated with 4 mg/kg i.v. JAQ1 also underwent transient thrombocytopenia (Supplementary Fig. 3a). Flow cytometric and Western blot analysis revealed that while mGPVI was completely downregulated, hGPVI was only reduced by 52.7 ± 16.4% in circulating platelets relative to control IgG-treated *hGP6*^*wt/tg*^ mice (Supplementary Fig. 3b–g). Detection of anti-rat IgG^FITC^ antibody binding confirmed rapid downregulation of the receptor rather than its opsonization (Supplementary Fig. 3h). Notably, we could further confirm that *hGP6*^*wt/tg*^ mice showed a homogeneous population of hGPVI^LO^ platelets after JAQ1 treatment (Supplementary Fig. 3i).

Next, we treated *hGP6*^*wt/tg*^ mice with 4 mg/kg i.v. of either Emf1 or Emf2 which induced a transient thrombocytopenia in both groups (Supplementary Fig. 3j, k). As expected, Emf1 and Emf2 caused rapid and sustained downregulation of hGPVI (Supplementary Fig. 3l, m), whereas the mGPVI receptor population was unaffected by the treatment, indicating that only those receptors that are directly targeted by the antibodies are downregulated (Supplementary Fig. 3n, o). Western blot analysis on day 5 after Emf1 treatment further confirmed complete depletion of hGPVI, but not mGPVI, from the circulating platelets (Supplementary Fig. 3p–s). Altogether, these data show that even though *hGP6*^*wt/tg*^ mice express only 50% of hGPVI, JAQ1 still only depleted half of these receptors, demonstrating that this effect is independent of receptor density.

### Antibody affinity for GPVI determines the degree of receptor downregulation in vivo

We next sought to understand the molecular mechanisms underlying the generation of hGPVI^LO^ platelets by JAQ1 and suspected that its affinity for hGPVI could have a role. Indeed, bio-layer interferometry (BLI) revealed that JAQ1 binds mGPVI with a K_D_ of 0.435 nM (Supplementary Fig. 4a, b), but hGPVI only with a K_D_ of 9.60 nM (Supplementary Fig. 4c, d). This marked difference in affinity for mGPVI vs. hGPVI may, at least in part, also explain why the antibody potently inhibits the receptor in *WT* mice, but not in human platelets.^34^ Of note, Emf1 bound to hGPVI with high affinity (K_D_ of 0.490 nM) (Supplementary Fig. 4e, f), very similar to that of JAQ1 for mGPVI. Based on this data, we speculated that the low affinity of JAQ1 for hGPVI was responsible for the incomplete depletion of the receptor in vivo. To test this hypothesis, we assessed the in vivo effects of the anti-mGPVI antibody JAQ4, which binds the mouse receptor with low affinity (K_D_: 21 nM) (Supplementary Fig. 4g, h).

Treatment of *WT* mice with 4 mg/kg JAQ4 IgG caused transient thrombocytopenia and a partial downregulation of mGPVI, with a drop to ~46.3% between 24 h and 5 days after injection (Supplementary Fig. 5a, b), which was also confirmed by Western blot analysis (Supplementary Fig. 5c, d). Finally, in-depth flow-cytometric analysis confirmed that JAQ4 treatment recapitulated the GPVI^LO^ phenotype in *WT* mice by generating low-density mGPVI platelets in circulation (Supplementary Fig. 5e). Similarly to hGPVI^LO^ platelets, these mGPVI^LO^ platelets showed abolished response to CRP, while the response to CVX was reduced. As expected, thrombin-induced activation and aggregation was unaltered in these mice (Supplementary Fig. 5f, g). Furthermore, collagen-induced platelet aggregation was impaired, but not abolished (Supplementary Fig. 5h, i). Finally, mGPVI^LO^ platelets displayed significantly reduced adhesion and aggregate formation in a whole blood perfusion assay on collagen (200 µg/mL) under arterial shear rates (1000 s^−1^) (Supplementary Fig. 5j–l). In conclusion, JAQ4 treatment generated a mGPVI^LO^ phenotype in *WT* mice, thereby strongly suggesting that low affinity anti-GPVI IgGs may generally have only the potential to induce partial immunodepletion of the receptor in vivo.

### Strong antithrombotic protection and diminished LPS-induced ALI but intact hemostasis in hGPVI^LO^ mice

**Fig. 5:**
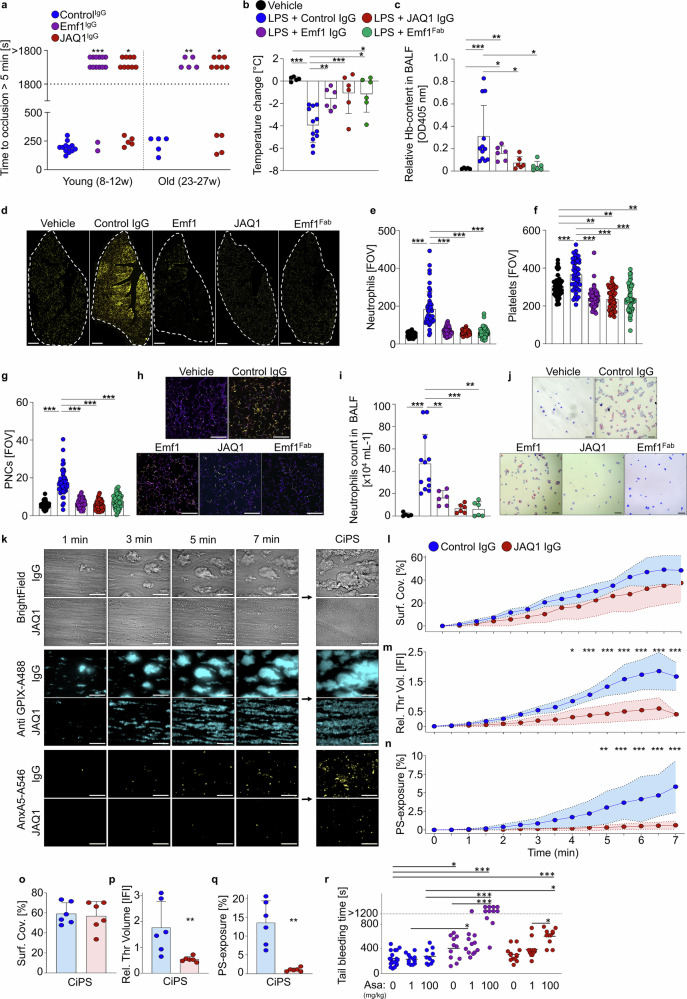
hGPVI^LO^-phenotype confers thrombo-protection without worsening ASA-associated bleeding. **a** Arterial thrombosis assessed 5 d post treatment (Emf1, JAQ1, or control IgG) using an aortic injury model in young and aged mice (male and female). **b**–**h** LPS-induced ALI: changes in body temperature (**b**), BALF hemoglobin (Hb) content (**c**), neutrophil infiltration (**d**, **e**), platelet (**f**) and PNC counts (**g**); whole lung tile-scans (scale bar: 1 mm) (**d**) and representative FOV (scale bar: 50 µm) (**h**) are shown. **i**, **j** Neutrophil quantification in BALF (**i**) and representative images (**j**). Analysis of neutrophil, platelet and PNC numbers per FOV (**e**–**g**), each dot representing one FOV (*n* = 5). **k**–**q** Flow-based analysis of platelet adhesion (**l**, **o**), aggregate formation (**m**, **p**) and (Phosphatidylserine) PS-exposure (**n**, **q**) over time on Horm collagen (200 µg/mL) under flow (1000 s^−1^) in heparinized blood from adult (23–27 weeks), 3 male and 3 female mice *hGP6*^*tg/tg*^ mice before (**l**–**n**) and after (**o**–**q**) Ca^2+^ perfusion; **k** representative images are shown; scale bar: 50 µm; CiPS = Calcium-induced Procoagulant State; AnxA5 = Annexin A5. **r** Tail bleeding assay after ASA and antibody treatments. Statistics: Fisher exact test (**a**), Kruskal–Wallis test (**b**, **c**, **e**–**g**, **i**), two-way ANOVA followed by Bonferroni post hoc testing (**l**–**n**, **r**) or Mann-Withney U test (**o**–**r**). Data are expressed as mean ± SD; significance is expressed as *p < 0.05, **p < 0.01, ***p < 0.001, vs. indicated group

To compare the impact of hGPVI^KO-like^ and hGPVI^LO^ phenotypes on thrombosis and hemostasis, we first applied a model of arterial thrombosis where the abdominal aorta is mechanically injured and the blood flow/occlusive thrombus formation is monitored by an ultrasonic flow probe.^35^ While 100% (12/12) of the control IgG-treated *hGP6*^*tg/tg*^ animals developed stable vessel occlusion, Emf1-treated (hGPVI^KO-like^) animals were potently protected from thrombotic vessel occlusion with ~85.7% (12/14) not forming a stable thrombus within the observation period of 30 min, whereas ~64.3% (9/14) of the animals were protected in the JAQ1-treated group (Fig. 5a). These protective effects were also evident in older animals (~27 weeks old; Fig. 5a). Notably, hGPVI^KO-like^ animals showed only transient reductions of blood flow, while hGPVI^LO^ mice developed short-lived, complete occlusions followed by rapid recanalization, indicative of reduced thrombus stability (Supplementary Fig. 6a). In comparison, the GPVI-blocking agent Emf6.1^Fab^ was previously shown to protect against thrombosis in the same model at a dose of 4 mg/kg after injection, however it provided only short-lived protection due to its limited half-life, with efficacy largely lost within 48 h after injection.^16^

A previous study from our group demonstrated that the hGPVI^KO-like^ phenotype in *WT* mice results in reduced LPS-induced ALI with improved physiological outcome.^8^ To test the efficacy of the GPVI^LO^ phenotype in this setting, we challenged Emf1-, JAQ1- or control IgG-treated *hGP6*^*tg/tg*^ mice with LPS 10 mg/kg intranasally (IN). As a control, mice were treated with Emf1^Fab^ which functionally blocks GPVI-induced platelet activation but does not affect GPVI surface expression. Notably, while all treatments led to a reduced temperature drop 4 h after LPS treatment (Fig. 5b), hGPVI^LO^ and Emf1^Fab^ treatment reduced hemoglobin content in the broncho-alveolar lavage fluid (BALF) (Fig. 5c). Finally, immunofluorescence mircoscopy of lung sections revealed markedly reduced neutrophil and platelet infiltration in the lungs (Fig. 5d–h), associated with marked reduction of neutrophil extravasation into the alveolar space for all tested conditions (Fig. 5i, j).

To elucidate the mechanisms underlying the strong protective effect of the hGPVI^LO^ phenotype in vivo, we analyzed platelet aggregate formation on collagen under flow. Old *hGP6*^*tg/tg*^ mice (23–27 weeks) were treated with JAQ1 or control IgG (4 mg/kg b.w.) and on day 5 heparinized whole blood was perfused for 7 min at a shear rate of 1000 s^−1^ followed by perfusion with Tyrode’s containing 2 mM Ca^2+^ for 5 min to induce a Calcium-induced Procoagulant State (CiPS). Under these experimental conditions, adhesion of hGPVI^LO^ platelets to collagen was unaltered compared to control (Fig. 5k, l), whereas thrombus formation was impaired (Fig. 5k, m) and phosphatidylserine (PS)-exposure, a marker of procoagulant activity, was virtually abolished over the entire observation period (Fig. 5k, n). Perfusion of Ca^2+^ over the adhered platelets led to a marked increase in PS-exposure from control IgG-treated mice, whereas this did not occur in hGPVI^LO^ samples (Fig. 5k, q). Concomitantly, while thrombi from control mice remained stable, marked thrombus disaggregation was observed in the hGPVI^LO^ samples, whereas platelet adhesion to collagen remained unaltered (Fig. 5k, o, p). Similarly, at higher shear rates (1700 s^−1^), hGPVI^LO^ platelets showed only slightly reduced adhesion to collagen, whereas thrombus formation and PS-exposure were again markedly impaired (Supplementary Fig. 6b–h).

Next, we assessed the impact of the hGPVI^KO-like^ and hGPVI^LO^ phenotype in a tail bleeding assay. As previously reported for mGPVI^KO-like^ as well as *Gp6*^*-/-*^ mice and *GP6*^*-/-*^ humans,^10,36,37^ hGPVI^KO-like^ mice displayed a small but significant increase in bleeding times. In contrast, bleeding times in hGPVI^LO^ mice remained unaltered compared to control. While the complete loss of GPVI alone only moderately impairs hemostasis, it can result in severely prolonged bleeding when combined with other antiplatelet agents, notably high dose ASA (100 mg/kg).^25^ We therefore assessed tail bleeding times in hGPVI^KO-like^ and hGPVI^LO^ mice treated with low (1 mg/kg) and high (100 mg/kg) dose ASA. In line with the previous reports, hGPVI^KO-like^ mice treated with 100 mg/kg ASA showed a severe hemostatic defect with 8/11 (~ 72.7%) animals being unable to arrest bleeding within the 20 min observation period. In sharp contrast, high dose ASA treatment only led to slight increase in bleeding times in hGPVI^LO^ mice and all of them stopped bleeding (Fig. 5r). Notably, a similar effect was seen in *hGP6*^*tg/tg*^ mice treated with Emf1^Fab^ in combination with 100 mg/kg ASA (Supplementary Fig. 6i), thus indicating that GPVI adhesive function significantly contributes to the hemostatic function.

### The signaling-dependent GPVI downregulation mechanism is conserved across species

**Fig. 6:**
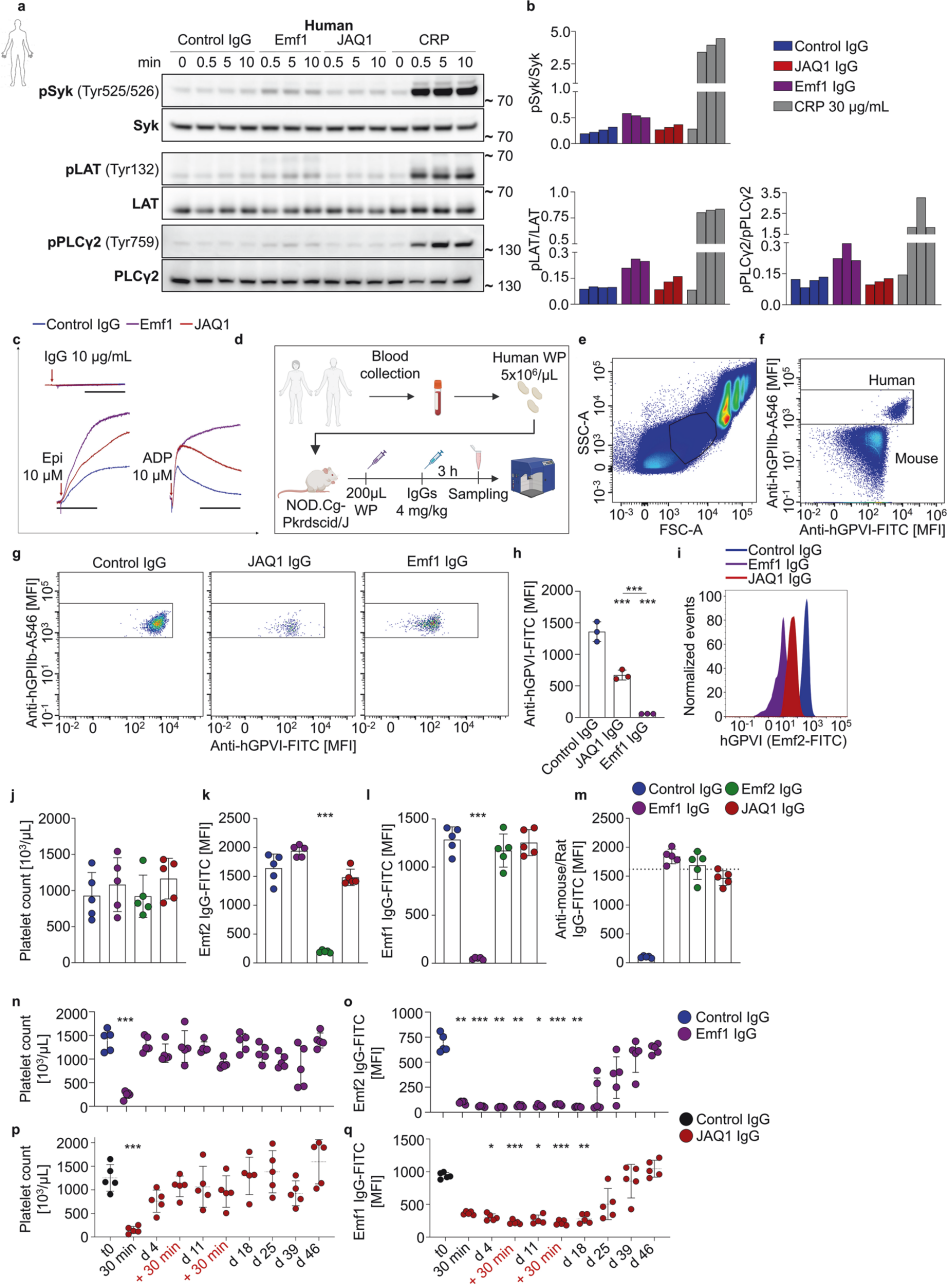
GPVI downregulation is mediated via a conserved signaling mechanism. **a**, **b** Western blot analyses of Syk, LAT and PLCγ2 phosphorylation in human washed platelets (**a**, **b**) platelets stimulated with Emf1, JAQ1, control IgG (20 µg/mL) or with CRP (30 µg/mL) as control. Western blot bands (**a**) and relative quantifications (**b**) are shown. **c** Aggregation traces in response to the indicated IgGs with or without low-dose epinephrine (10 µM) or ADP (5 µM). Scale bar: 5 min (vehicle and ADP) or 10 min (Epinephrine). **d**–**i** NOD/SCID mice (*n* = 3) transfused with human platelets followed by antibody injection; experimental scheme (**d**), gating (**e**, **f**), post-treatment GPVI expression (**g**, **h**) and population profiling (**i**). **j**–**m** *hGP6*^*tg/tg*^ mice (*n* = 5) pre-treated with 2.4G2 (4 mg/kg b.w.) followed by administration of Emf1, Emf2, JAQ1, or control IgG (4 mg/kg b.w). **j** Platelet counts were determined using an automated cell counter. **k**, **l** Surface hGPVI expression was assessed by flow cytometry using Emf2-IgG^FITC^ (**k**) or Emf1-IgG^FITC^ (**l**). **m** Receptor occupancy was evaluated using anti-mouse IgG^FITC^ (for Emf1 and Emf2) or anti-rat IgG^FITC^ (for JAQ1). Dashed lines indicate in vitro opsonization controls obtained by incubating whole blood with 50 µg/mL antibody. **n**–**q** Repetitive injection of Emf1 (**n**, **o**) or JAQ1 (**p**, **q**) in *hGP6*^*tg/tg*^ mice: platelet count and GPVI surface levels were assessed over time. Statistics: Kruskal–Wallis test (**h**, **j**–**m**) or the Friedman Test (**n**–**q**). Data are expressed as mean ± SD; significance is expressed as *p < 0.05, **p < 0.01, ***p < 0.001, vs. indicated group

Previous studies demonstrated that the antibody-induced GPVI downregulation in vivo strictly depends on ITAM signaling downstream of receptor.^38^ We therefore suspected that the degree of GPVI downregulation may be influenced by the strength of signaling induced by the GPVI targeting mAb. To test this directly, we treated washed platelets from healthy human donors with 20 µg/mL Emf1, JAQ1, or control IgG in vitro and assessed changes in tyrosine phosphorylation of key signaling proteins downstream of GPVI (Syk, LAT, and PLCγ2). Notably, both Emf1 and JAQ1 induced detectable ITAM signaling, which was, however, consistently weaker with JAQ1 compared to Emf1 (Fig. 6a, b). Importantly, phosphorylation patterns were comparable between murine and human platelets, as shown by Western blot analyses of Syk and LAT after pre-incubation of *hGP6*^*tg/tg*^ platelets under the same conditions (Supplemental Fig. 7a, b), indicating that a conserved signaling-dependent mechanism drives GPVI downregulation. Consistent with this, Emf1 or JAQ1 alone did not trigger platelet aggregation in human and *hGP6*^*tg/tg*^ platelet-rich-plasma (PRP). However, both antibodies potentiated platelet aggregation in response to low-dose epinephrine (10 µM) and ADP (5 µM) in line with previous observations in mouse platelets,^39^ with Emf1 showing a markedly stronger effect than JAQ1, again reflecting a difference in signaling strength (Fig. 6c and Supplementary Fig. 7c).

To directly test whether human platelets are susceptible to antibody-induced GPVI downregulation in vivo, we transfused platelets from healthy volunteers into immuno-deficient NOD/SCID mice, which permit extended circulation of human cells.^23^ Subsequent treatment with Emf1 or JAQ1 led to ~100% and ~48% depletion of hGPVI, respectively, from the transfused human platelets in vivo (Fig. 6d–i), mirroring the effects observed in the *hGP6*^*tg/tg*^ mouse model.

To confirm whether GPVI depletion by Emf1, Emf2, and JAQ1 would occur through a similar mechanism in vivo, we pre-treated *hGP6*^*tg/tg*^ mice with the anti-FcγRIIa/b antibody 2.4G2 and observed abolished platelet recruitment to LSECs (Supplementary Fig. 7d) and, as a consequence, neither transient thrombocytopenia nor GPVI depletion, although the circulating platelets were fully opsonized with the antibodies (Fig. 6j–m). Finally, given the therapeutic potential of partial GPVI downregulation, we repeatedly injected JAQ1 and Emf1 in *hGP6*^*tg/tg*^ or JAQ4 in *WT* mice and monitored the platelet phenotype over time. Very consistently, the second and third injections neither caused thrombocytopenia nor a further reduction in receptor abundance, but maintained the GPVI^LO^ and GPVI^KO-like^ phenotype over extended time periods (Fig. 6n–q and Supplementary Fig. 7e, f).

## Materials and methods

### Animals

Animal experiments received approval from the District Government of Lower Franconia (Regierung von Unterfranken) and were conducted following the guidelines outlined in the Animal Research: Reporting of In Vivo Experiments (ARRIVE) (https://arriveguidelines.org/). The mice were matched by age, sex, and genetic background. To exclude possible confounders due to the sex, both male and female mice were used for all experiments. The humanized *GP6* mouse line (*hGP6*^*tg/tg*^)^34^ and the NOD.Cg-Pkrdcscid/J (Charles River, France) were described before.^23^

### Blood donors and blood collection

Blood was collected from healthy volunteers who had not been on anticoagulant or anti-platelet therapy for at least four weeks. Blood samples were obtained after written informed consent in accordance with the Declaration of Helsinki and approval by the Institutional Review Boards of the University of Würzburg (approval number: 295/20). Blood was drawn by venipuncture using butterfly needles and collected into 9 mL tubes containing 3.2% trisodium citrate. For all studies, the blood was kept at room temperature and used within four hours. All methods were performed in accordance with the relevant guidelines and regulations.

### Mechanical injury of the abdominal aorta

In anesthetized mice (male and female 10–12 weeks old or 23–27 weeks old), the aorta was exposed and a Doppler ultrasonic flow probe was positioned around the vessel. Vascular injury was induced by clamping the aorta for 5 s. Blood flow was then monitored for 30 min or until complete occlusion was observed, defined as a cessation of blood flow for more than 5 min.

### Statistics

Statistical analyses were performed using GraphPad Prism version 9.0.0 (GraphPad Software). The Shapiro–Wilk test was used to assess data distribution. Based on the distribution, group differences were evaluated using either the Mann–Whitney U test, the Kruskal–Wallis test for one-way analysis, or two-way ANOVA followed by Bonferroni post hoc testing. Fisher’s exact test was applied for categorical data analysis. A threshold of *P* ≤ 0.05 was considered statistically significant, and results are presented as mean ± standard deviation. A detailed description of all materials and methods used in this study is provided in the supplements.

## Discussion

Our data demonstrate for the first time that pharmacological downregulation of platelet surface GPVI by low-affinity antibodies is feasible and induces a stable GPVI^LO^ phenotype. This partial depletion is sufficient to confer robust antithrombotic and antithrombo-inflammatory protection without increasing bleeding risk, in contrast to full GPVI depletion or genetic GPVI deficiency.^10,35^

GPVI is an attractive therapeutic target due to its exclusive expression on megakaryocytes and platelets, which minimizes off-target effects.^10,11^ Inherited GPVI deficiency in humans is typically associated with only mild bleeding symptoms,^37,40^ underscoring its dispensability for normal hemostasis. Notably, GPVI was the first platelet receptor shown to be depletable in vivo via antibody-mediated mechanisms, introducing a new concept for antiplatelet therapy.^10^

Due to their anucleate state and minimal protein synthesis, platelets are particularly amenable to irreversible inhibition. This principle underlies the long-lasting effects of established agents such as acetylsalicylic acid (ASA) and clopidogrel, which irreversibly inhibit COX and P2Y₁₂, respectively. Among platelet adhesion receptors, only GPVI and CLEC-2 contain (hem)ITAM motifs and are susceptible to irreversible antibody-induced silencing. However, CLEC-2 inhibition raises safety concerns given its critical role in maintaining blood-lymphatic separation^41,42^ and its targeting has been associated with adverse events such as cerebral venous thrombosis.^43^ In contrast, GPVI inhibition or depletion has consistently shown strong efficacy in preclinical models of thrombosis and thrombo-inflammation without causing major bleeding complications.^8–10,16,44^

This likely reflects redundancy in platelet activation pathways. In normal hemostasis, platelets are activated by multiple exposed or released agonists, including thrombin, ADP, TxA₂ and others which are sufficient to trigger clot formation even in the absence of functional GPVI. This redundancy probably explains the mild bleeding phenotype in GPVI-deficient humans and mice,^10,37,40^ and why bleeding risk becomes significant only when additional pathways are inhibited (e.g., with high dose ASA).

Several pharmacological strategies have been explored to target GPVI. Among them, monovalent Fab fragments that block signaling while preserving adhesion have shown promise,^16^ but their short in vivo half-life limits their utility for long-term treatment. In contrast, IgG-mediated immunodepletion offers a more sustained approach. Clinical observations in patients with immune thrombocytopenia (ITP) and chronic anti-GPVI autoantibodies support the concept that long-term GPVI downregulation can occur in humans through similar mechanisms as in mice.^30^

Mechanistically, receptor depletion involves Fcγ receptor IIB-dependent platelet recruitment to the liver, where the IgG-opsonized GPVI clusters on liver sinusoidal endothelial cells (LSECs) leading to its depletion.^22^ This process depends on GPVI signaling, as mice expressing a signaling-defective FcRγ-chain variant fail to undergo IgG-mediated depletion of the receptor.^38^ Our study now reveals that high-affinity binding is essential for complete GPVI depletion. In contrast, low-affinity antibodies reproducibly induced only partial downregulation (~50%), suggesting a signaling threshold is required for full immunodepletion. Based on these data, we speculate that low-affinity antibodies fail to induce sufficient receptor clustering and downstream signaling to engage the FcRγ-ITAM signaling pathway effectively. This is further supported by our data showing that Emf1 and JAQ1 trigger GPVI-signaling with different strengths, a phenotype conserved between human and mouse platelets. These findings suggest that antibody affinity can be exploited to fine-tune the extent of receptor downregulation, providing a novel strategy for graded, safe long-term antiplatelet therapy. Notably, this aligns with previous reports that tetravalent GPVI ligands are required for robust platelet activation, whereas divalent or trivalent ligands elicit minimal signaling insufficient to trigger aggregation.^45^

In translating these findings to human therapy, the expression of FcγRIIA (CD32) on human—but not murine—platelets could raise concern. However, our data argue against a functional contribution of FcγRIIA in this setting. Western blot analysis revealed similarly subtle intracellular signaling in human and murine platelets upon anti-GPVI antibody binding, suggesting that FcγRIIA was not activated (Fig. 6a, b and Supplementary Fig. 7a, b). This is consistent with the reported low affinity of FcγRIIA for monomeric IgG, which typically requires clustered immune complexes or surface-immobilized antibodies for activation. Moreover, transfusion of human platelets into NOD/SCID mice confirmed that both complete (Emf1) and partial (JAQ1) receptor downregulation is achievable in human platelets in vivo (Fig. 6d–f), indicating that the underlying Fc-dependent mechanism is conserved across species. Importantly, blocking FcγRIIB with 2.4G2 prevented thrombocytopenia and receptor loss in *hGP6*^*tg/tg*^ mice, supporting the established role of FcγRIIB-expressing LSECs this process.^22^ These findings collectively suggest that FcγRIIA is not involved in GPVI downregulation and underscore the translational potential of this approach.

Finally, data from patients with GPVI autoantibodies—as seen in immune thrombocytopenia (ITP)—further support the clinical relevance of this mechanism.^26–30^ In these cases, pronounced and often prolonged GPVI loss from the platelet surface has been observed. In contrast to this uncontrolled immune response, we show that after reversal of the initial transient platelet sequestration and return of the GPVI-depleted platelets to circulation, repeated administration of Emf1 or JAQ1 does not induce renewed thrombocytopenia, while maintaining stable receptor downregulation. This demonstrates the feasibility of sustained GPVI-targeted therapy for the prevention of arterial thrombosis and thrombo-inflammatory conditions.

The hGPVI^LO^ phenotype reveals a mechanistic uncoupling of platelet adhesion to collagen from GPVI-dependent signaling and PS exposure, thereby enabling effective antithrombotic protection with minimal bleeding risk. These findings align with our previous data showing that GPVI blockade by Emf6.1^Fab^ selectively impairs signaling while preserving adhesion.^16^ Together, these findings suggests that residual GPVI-mediated adhesion is sufficient to maintain vascular integrity and hemostasis, whereas intracellular GPVI signaling is a key driver of arterial thrombosis and thrombo-inflammation. These insights highlight the therapeutic value of selectively modulating GPVI signaling rather than ablating the receptor entirely. This concept is further supported by observations in the model of LPS-induced pulmonary thrombo-inflammation and resultant inflammatory bleeding. In this model, hGPVI^KO-like^ mice displayed markedly reduced neutrophil recruitment and extravasation, but showed unaltered vascular leakage, as indicated by unchanged hemoglobin content in BALF compared to LPS-treated control. In contrast, both hGPVI^LO^ and Emf1^Fab^-treatment significantly reduced BALF hemoglobin content, pointing to improved vascular barrier function under inflammatory stress. These results support the idea that partial GPVI depletion or signaling inhibition reduces inflammation-induced tissue injury while preserving endothelial integrity.

LPS inhalation in mice mimics key features of acute lung injury (ALI) and acute respiratory distress syndrome (ARDS) which are associated with high morbidity and mortality in humans.^46^ ARDS is characterized by intense inflammation, increased vascular leakage, pulmonary edema, and alveolar bleeding, ultimately leading to severely impaired gas exchange, a process that has recently been shown to be exacerbated by platelet-derived integrin and tetraspanin-enriched tethers (PITTs).^47^ In this context, full GPVI inhibition (e.g., Emf1^Fab^, acute) or partial GPVI depletion (hGPVI^LO^, sustained treatment) may limit both edema formation and bleeding, offering new therapeutic options for thrombo-inflammatory lung injury.

Importantly, GPVI plays a critical role in limiting blood loss during inflammation,^48,49^ and in malignancy-associated coagulopathies.^44^ In such settings, partial GPVI downregulation may be preferable to complete receptor depletion, as it would reduce thrombotic risk while preserving essential hemostatic functions. This is consistent with prior studies showing that even moderate reductions in GPVI surface levels significantly alter platelet responsiveness to collagen and shift thrombosis thresholds in vivo.^34^ Furthermore, GPVI surface expression is known to be tightly regulated, supporting the concept that a critical receptor density is required for optimal platelet function.^15^

Prolonged antiplatelet therapy remains a cornerstone in the prevention of arterial thrombosis, ischemic stroke, and myocardial infarction.^50^ Current strategies include single antiplatelet therapy (SAPT) with ASA or dual antiplatelet therapy (DAPT) combining ASA with P2Y_12_ inhibitors such as clopidogrel or ticagrelor. While DAPT provides enhanced protection, it is associated with increased bleeding risk during long-term use.^50^ In this context, targeting membrane glycoproteins such as GPVI to modulate their surface abundance may offer a novel therapeutic strategy to balance antithrombotic efficacy with improved safety.

Notably, IgG-based antibodies exhibit long in vivo half-lives, allowing for sustained antithrombotic effects with reduced dosing frequency. In our study, repeated administration of Emf1 or JAQ1 extended GPVI downregulation for up to 41 and 25 days respectively, without reducing platelet counts as the circulating (already GPVI-depleted) platelet population is not susceptible to antibody-induced sequestration or removal as described previously.^10^ Although newly produced platelets expressing intact GPVI continuously enter the circulation, they represent only a small fraction of the total platelet pool at a given time that is only transiently sequestered and then also return to circulation. A key limitation of this approach remains the transient thrombocytopenia observed after the initial dose, which could potentially be mitigated by gradual dosing strategies or slow-release formulations, thereby reducing the initial impact on the circulating platelet pool. This may be particularly important for patients with pre-existing thrombocytopenia or elevated bleeding risk.

In summary, we hereby demonstrate that antibody-mediated downregulation of hGPVI represents a feasible and scalable approach for in vivo receptor silencing. High-affinity antibodies induce complete GPVI depletion, whereas low-affinity variants generate a stable GPVI^LO^ phenotype characterized by reduced thrombus formation but preserved collagen adhesion. This intermediate phenotype may provide a safer alternative to complete GPVI inhibition, especially for long-term antithrombotic therapy, including combination regimens with ASA.

## Supplementary information

Supplemental material revised QC

## Data Availability

The raw data supporting the findings of this study have been deposited in the RADAR research data repository of the University of Würzburg and are publicly available: https://wuedata.uni-wuerzburg.de/radar/en/search (10.58160/3bueyhbaxn4735vh).
