# Supplementary material for: Partial downregulation of platelet glycoprotein VI by low affinity antibodies confers sustained and safe antithrombotic protection
Source: Signal Transduct Target Ther. 2026 Jul 1;11:264. doi: 10.1038/s41392-026-02864-5 (PMC13323380; doi:10.1038/s41392-026-02864-5)
Supplement: Supplementary file 1 — Supplemental material revised QC [file 41392_2026_2864_MOESM1_ESM.docx]

Supplementary Materials for

Partial downregulation of platelet glycoprotein VI by low affinity antibodies confers sustained and safe antithrombotic protection

Stefano Navarro^1, 2^, Sarah Beck^1,2^, Sabrina I. Bonfiglio^1,2^, Ernesto J. Cuenca-Zamora^1,2^, Lukas J.Weiß^1,3^, Marijke Kuijpers^4^, Johan Heemskerk^5^, David Stegner^1,2^, Bernhard Nieswandt^1,2^

^1^ Institute of Experimental Biomedicine I, Würzburg Josef-Schneider-Straße 2, 97080 Würzburg, Germany.

^2^ Rudolf Virchow Center; Center for Integrative and Translational Bioimaging; University of Wuerzburg Josef-Schneider-Str. 2, 97080 Würzburg Germany.

^3^ Department of Internal Medicine I University Hospital Würzburg

^4^ Department of Biochemistry, Cardiovascular Research Institute Maastricht (CARIM), Maastricht University, 6229 ER Maastricht, The Netherlands.

^5^ Synapse Research Institute, Kon. Emmaplein 7, 6214 KD Maastricht, The Netherlands

Corresponding author:

Bernhard Nieswandt, PhD

Institute of Experimental Biomedicine, University Hospital and

Rudolf Virchow Center, University of Würzburg

Josef-Schneider-Straße 2, 97080 Würzburg, Germany

Tel.: +49 931 31 80405; Fax: +49 931 60 80405.

E-mail address: [bernhard.nieswandt@uni-wuerzburg.de](mailto:bernhard.nieswandt@uni-wuerzburg.de)

This PDF file includes:

Materials and Methods

Figures S1 to S7

Table S1

**Materials and Methods**

Antibodies and Reagents

Horm collagen was purchased from Takeda (Linz, Austria); The collagen-related peptide (CRP) was purchased from Cambridge Research Biochemicals (Cambridge, UK); ADP, apyrase, prostacyclin (PGI_2_), fibrinogen and hematoxylin were from Sigma Aldrich (Steinheim, Germany). thrombin was purchased from Roche Diagnostic (Mannheim, Germany); convulxin was purchased from Enzo Life Sciences (New York, NY, USA rabbit anti-GAPDH and rat anti-mouse IgG-HRP antibodies were purchased from Sigma-Aldrich (Steinheim, Germany);; anti-rabbit IgG-HRP was purchased from Jackson Immuno (Suffolk, UK); goat anti-rat IgG-HRP was purchased from Dianova (Hamburg, Germany). The micro-cuvettes for aggregometry were purchased from LABITec (Ahrensburg, Germany). For the collection of human blood, S-monovettes 3.2% citrate and Safety-Fly-Needle 21G were purchased from Sarstedt (Nümbrecht, Germany). Heparin was purchased from Ratiopharm (Ulm, Germany); the 5 mL polystyrene round-bottom Tubes for flow cytometry were purchased from Corning Inc. (New York, NY, USA).^1^ Epinephrine was purchased from Selleckchem (Texas, USA). JAQ1,^2^ JAQ2,^3^ JAQ4 (unpublished), Emf1^1^, Emf2,^4^ JON/A^5^ and WUG 1.9^5^, 2.4G2 were produced, purified and derivatized in-house. Midazolam (Roche Pharma AG), Dormitor (Orion Pharma), and fentanyl (Janssen-Cilag GmbH) were used according to regulations of the local authorities. A more detailed description of the anti-GPVI antibodies used in this article is provided in supplemental table 1.

Washed murine platelets

Murine washed platelets were obtained as follows: Whole blood was collected in 20 U/mL heparin via retro-orbital bleeding following isoflurane anesthesia. Heparinized blood was centrifuged for 6 min at 300 *g*. The obtained PRP was supplemented with 2 µL apyrase and 5 µL PGI_2_ as for human platelets. Next, platelets were pelleted by centrifugation for 5 min at 800 *g*, washed twice with Tyrodes buffer (supplemented with 2 µL apyrase and 5 µL PGI_2_). Finally, the platelets were resuspended to 500.000/µL in tyrodes buffer and kept for 30 minutes at 37°C before use.

Washed Human platelets

Citrated human blood was collected in a 10 mL S-monovette and implemented with 2 mL of ACD pH 4.5 and then the sample was centrifuged for 20 min at 150 g at room temperature. Platelet-Rich-Plasma (PRP) was collected in new 15 ml tubes and supplemented with 1/10 ACD, 2 μL of apyrase/mL (0.02 U mL−1; A6410, Sigma-Aldrich) and 5 μL PGI_2_/µL (0.1 μg mL−1; P6188, Sigma-Aldrich). Platelets were pelleted by centrifugation for 10 min at 500 *g*, washed twice with Tyrode’s buffer (N-2-hydroxyethyl-piperazine-N02-ethanesulphonic acid; 134 mM NaCl, 0.34 mM NaH_2_PO_4_, 2.9 mM KCl, 12 mM NaHCO_3_, 5 mM HEPES, 5 mM glucose, 0.35% BSA, pH 7.4) containing 2 µL apyrase/mL and 5 µL PGI_2_/mL and finally resus- pended at a concentration of 500,000/µL in Tyrodes buffer and kept for 30 min at 37°C before use.

Aggregometry

Washed murine or human platelets were diluted in Tyrode’s buffer supplemented with 2 mM Ca^2+^ (50 µL platelet suspension/110 µL Tyrode’s buffer) and 100 μg/mL human fibrinogen. When thrombin was used as agonist, Tyrode’s buffer was not supplemented with fibrinogen. Light-transmission aggregometry was performed to follow platelet aggregation over time using a 4-channel APACT aggregometer (LABITec Ahrensburg, Germany) under stirring conditions for 10 min after pre-incubation with the antibodies and the addition of the specified agonists.

When indicated, PRP was used for the aggregometry assay. PRP from human and mouse were collected, pre-incubated as described and subsequently diluted in Tyrode’s containing 2 mM Ca^2+^ (without fibrinogen) in the same ratio as for the washed platelets.

Classic flow adhesion assay

200 μg/mL Horm collagen was coated on coverslips for 1 hour at 37°C and then blocked using 1% BSA in PBS. Heparinized murine blood (8-12 weeks old) was diluted 2:1 in Tyrode’s buffer and supplemented with 2 mM Ca^2+^. Platelets were labeled with an anti-GPIX-Dylight (Dy) 488 (p0p6) conjugated antibody.^6^ Blood was perfused over the coverslips at a shear rate of 1000 s^-1^ for 4 min and subsequently washed for 4 more minutes with Tyrode’s buffer supplemented with Ca^2+^. After washing, 8 representative fields of view were imaged using a Leica DMI6000B microscope with a 63x objective (Leica Biosystems Technologies, Frankfurt, Germany). Finally, images were analyzed for overall using Fiji.^7^ Fluorescence signals of the indicated labels were manually thresholded. Platelet adhesion to the surface was measured as percentage of area coverage of the anti-GPIX^Dy488^ fluorescent signal, while relative thrombus volume was analyzed based on its fluorescent integrated density using Fiji. Fluorescent integrated density is calculated in Fiji as the sum of all pixel intensities of the platelet-specific signal (anti-GPIX^Dy488^) within a defined thrombus area, which correlates both with the lateral expansion of the thrombus (area) and the accumulation of fluorescent signal in the *z*-direction (pile-up of platelets on top of each other), effectively providing a semi-quantitative estimation of thrombus volume.

Whole blood flow adhesion assay

The preparation of the slides was performed as described for the classic flow-adhesion assay. Heparinized, undiluted murine blood (23-27 weeks old) was perfused over the coverslips at a shear rate of 1000 s^-1^ or 1700s^-1^ for 7 and 4 min, respectively, and subsequently washed for 5 minutes with Tyrode’s buffer supplemented with 2 mM Ca^2+^. During the blood perfusion, images were taken every 30 s, after the wash 5 representative images were taken on a DMI6000B microscope using a 63x objective (Leica Biosystems Technologies, Frankfurt, Germany). Images were analyzed for overall platelet- (anti-GPIX^Dy488^), and phosphatidylserine (PS) (Annexin A5^AF546^) percentage of surface area coverage, while thrombus volume was assessed by analysing the fluorescent integrated density of the anti-GPIX^Dy488^ signal using Fiji as described above.^7^

Measurement of platelet count and size

To assess platelet count and size, mice were bled into EDTA-coated tubes; platelet parameters were measured using an automated cell counter (ScilVet, scil animal care company GmbH, Viernheim, Germany).

Flow cytometric analysis of GPVI expression and platelet activation

For the detection of GPVI expression, murine blood diluted 1:20 in Tyrode’s buffer without Ca^2+^ was pre-incubated 10 min with the antibodies. For the platelet activation analysis, the murine blood was diluted in Tyrode’s buffer with 2 mM Ca^2+^. JON/A-PE was used to detect the activated integrin αIIbβ3 whilst P-selectin exposure was used as marker for platelet degranulation and detected with a specific anti-mP-selectin^FITC^-conjugated antibody, WUG 1.9.^5^ The diluted murine blood was incubated with either CRP (10 µg/mL), thrombin (0.1 U/mL) or vehicle solution, together with JON/A-PE and Wug.E9^FITC^ for 12 min (6 min at 37°C and 6 min at RT). Finally, the reaction was stopped by diluting with 500 µL PBS and the samples subsequently measured on a FACSCelesta (BD Biosciences, Franklin Lakes, New Jersey, USA). The data were analyzed using the software FlowJo_v10.8.1.

Immunostaining of the liver to assess the mechanism of GPVI downregulation

*hGP6^tg/tg^* mice pretreated with 2.4G2 (4 mg/kg i.v.) or isotype control were subsequently treated with the anti-GPVI antibodies Emf1^AF488^, Emf2^AF488^, JAQ1^Dy488^ or control IgG^AF488^ (4mg/kg b.w.).One hour after injection the livers were harvested and placed in Tissue-Tek® Cryomold® (Sakura), submerged in O.C.T.TM Tissue Tek®, and snap frozen with liquid nitrogen. 7 µm sections were mounted onto Superfrost® Plus glass slides (Thermo Scientific). Rat anti-mouse GPIX^AF546^ (p0p6, 5 µg/mL) + anti-GPIbβ^AF546^ (p0p1 ,5 µg/mL), and rat anti-mouse CD31^AF547^ (10 µg/mL), diluted in blocking buffer were used to stain platelets and vessels, respectively. Sections were mounted using Fluoroshield™ with DAPI (Sigma Aldrich). Z-stacks of 661.59 x 661.59 µm were acquired using a Thunder Imager DMi8 (Leica Microsystems) equipped with a 63x objective; maximum projection is shown in the figures.

Tail bleeding assay

For the assessment of the tail bleeding time, *hGP6^tg/tg^* mice were treated i.v. with either Emf1, JAQ1 or control IgG 5 days prior to the experiment. In addition, mice were treated i.v. with either ASA 1mg/kg, 100mg/kg or vehicle. Following anesthesia (0.5 mg/kg Medetomidin, 5 mg/kg Midazolam und 0.05 mg/kg Fentanyl) of the animals, a scalpel was used to remove 2 mm tip of the tail. The tail bleeding was monitored by absorbing blood drops on filter paper with 20 s intervals avoiding contact between the paper and the wound. Bleeding was determined to have stopped when no blood could be absorbed from the paper. Experiments were stopped after 20 min. Differences between occluded and non-occluded wounds were statistically assessed using the Fisher’s exact test, the mean bleeding time of occluded vessels by Mann-U-Whitney test.

Assessment of antibody affinity

To investigate GPVI interactions, human or mouse GPVI-Fc fusion protein (1.2 µg) was immobilized on the biosensor using appropriate capture surfaces, and the binding of anti-GPVI antibodies was assessed via Bio-Layer Interferometry (BLI) on the Octet system. The resulting sensorgrams were analyzed using ForteBio's proprietary software. The assay parameters included an association phase of 900 seconds and a dissociation phase of 1200 seconds. Antigen screening was performed across seven concentrations as indicated in the graphs. A 1:1 interaction model was employed for fitting the kinetics data.

For the kinetic assays, hGPVI or mGPVI-Fc fusion protein (antigen)was first captured onto the biosensor surface. The biosensors with immobilized antigen were then introduced to wells containing varying concentrations of Emf1, JAQ1, or JAQ4 (association phase), followed by immersion in running buffer for the dissociation phase. To account for baseline drift, reference corrections were performed by capturing antigen and dipping the biosensors into wells containing only buffer. This allowed for compensation of natural antigen dissociation from the biosensor. All experiments were conducted at 25°C with a constant agitation speed of 1,000 rpm. A fresh biosensor was used for each sample. Dissociation rate constants (KD) were determined using ForteBio Data Analysis software, and all consumables utilized in the experiments were those recommended by ForteBio.

LPS-induced lung injury

Mice, under 2% isoflurane anesthesia, were intranasally instilled with LPS (Escherichia coli; O111:B4 serotype; Sigma Aldrich) at a dose of 10 mg/kg body weight (bw) to induce an acute alveolar inflammation. Sodium chloride (NaCl; 0.9%) served as vehicle control. After 4 hours, mice were anesthetized by intraperitoneal injection of ketamine (80 mg/kg bw) and xylazine (16 mg/kg bw). Mice were euthanized via exsanguination, and a bronchoalveolar lavage was performed through 3 washes, each with 600 μL PBS. The bronchoalveolar lavage fluid (BALF) was pooled, total cell counts were determined with a Neubauer improved hemocytometer, and cytospin preparations (Shandon Cytospin 4; Thermo Fisher Scientific) generated and stained with DiffQuick staining kit (HAEMA, LTSYS; Labor+ Technik, Eberhard Lehmann GmbH) for differential cell counts. For the immunostaining of the lung sections, the trachea was cannulated with a 22 G venous catheter (BD Insyte) and 800 µL of a 1:1 mixture of O.C.T.TM Tissue Tek® (Sakura) and 10% (w/v) sucrose in PBS was slowly injected to inflate the lungs. The trachea was tied up, the lung harvested *en-bloc* and placed in Tissue-Tek® Cryomold® (Sakura), submerged in O.C.T.TM Tissue Tek®, and snap frozen with liquid nitrogen. 7 µm sections were mounted onto Superfrost® Plus glass slides (Thermo Scientific). Rat anti-mouse GPIX^AF488^ (p0p6, 5 µg/mL) + anti-GPIbβ^AF488^ (p0p1, 5 µg/mL), rat anti-mouse Ly6G^AF647^ (2 µg/mL, 1A8, BioLegend) and rat anti-mouse CD31^AF546^ (10 µg/mL), diluted in blocking buffer were used to stain platelets, neutrophils and vessels, respectively. Sections were mounted using Fluoroshield™ with DAPI (Sigma Aldrich). Z-stacks of 10 fields of view (FOV) with 661.59 x 661.59 µm per section randomly distributed over the left lung were acquired using a Thunder Imager DMi8 (Leica Microsystems) equipped with a 20x objective (HC PL APO 20x/0.80 DRY, Leica Microsystems) and Leica-DFC9000GT-VSC12293 camera (Leica Microsystems). Maximum projection was used to count neutrophils, platelets, PNCs, manually using Fiji (Image J 2.3.0). A total of 10 FOV per lung were analyzed.

For the hemoglobin measurement, cell in the BALF were pelleted by centrifugation at 400 x g for 10 min at 4 °C. The cell pellet was resuspended in 100 µL ice cold ammonium-chloride-potassium (ACK) lysis buffer and incubated for 3 min on ice. Afterwards, the cell suspension was centrifuged at 400 x *g* for 10 min at 4 °C and 50 µL of the supernatant were transferred twice into wells of a 96-well plate. Spectrophotometric measurement was performed on a Spark multimode microplate reader (Tecan Trading AG) at 405 nm.

Body temperature was measured using an infrared thermometer (ETEKCITY Lasergrip 774, ETEKCITY Corp) at the abdomen of the animals before and 4 h after LPS or NaCl instillation. The difference in body temperature was calculated and is depicted in °C.

Western blot analysis of protein phosphorylation

Washed human or murine platelets (7 × 10⁵/µL) were prepared as described above (exception: no BSA in Tyrode’s buffer) and allowed to rest for 10 min at 37°C in Tyrode’s buffer without BSA. Platelets were preincubated with indomethacin (10 µM final concentration), apyrase (2 U/mL, high dose), and EDTA (5 mM) for 5 min at 37°C. Subsequently, 210 µL aliquots of the suspension were transferred into aggregometry cuvettes under stirring conditions, and 50 µL samples were collected before stimulation (0 s, resting) into an equal volume of ice-cold lysis buffer (300 mM NaCl, 20 mM Tris, 2 mM EGTA, 2 mM EDTA, pH 7.5) supplemented with 2% IGEPAL CA-630, 20 µL protease inhibitor cocktail, and 40 µL sodium orthovanadate. Platelet activation was triggered with the indicated agonists (CRP, 30 µg/mL; Emf1, JAQ1, control IgG, 20 µg/mL). At the indicated time points (30 s, 5 min, and 10 min), 50 µL samples were removed and lysed as above, centrifuged at 14,000 rpm for 10 min at 4°C, and supernatants were stored at -80°C until analysis. For SDS-PAGE, 45 µL of each lysate was mixed with 15 µL of 4× NuPAGE LDS sample buffer containing 2.5 µL β-mercaptoethanol, incubated for 10 min at 70°C, and resolved on 4–12% Bis-Tris gradient gels (Invitrogen) at 4°C. Proteins were transferred onto PVDF membranes using a semi-dry blotting system at 50 mA for 50 min. Membranes were blocked in 5% BSA in TBS-T for 1 h at room temperature and probed overnight at 4°C with anti-phosphotyrosine (clone 4G10, 1:1000, Millipore, Cambridge, UK) or the specific indicated antibodies (Syk #12358 Cell Signaling, Cambridge, USA; pSyk #2711 Cell Signaling; pLAT #ab4476 Abcam; LAT #9166 Cell Signaling; pPLCγ2 #3874 Cell Signaling; PLCγ2 #3872 Cell Signaling) in 5% BSA/TBS-T containing 0.1% sodium azide. After washing (3×10 min in TBS-T), membranes were incubated for 1 h with HRP-conjugated anti-mouse secondary antibody (#115-035-166 Jackson Immunoresearch, Pennsylvania, USA) for 4G10 or anti-rabbit-HRP (#7074 Cell Signaling), washed three times in TBS-T, and developed using enhanced chemiluminescence (ECL, Perkin Elmer, Massachusetts, USA).

Transfusion of NOD/SCID mice with human platelets

Citrated human blood from 3 healthy volunteers was collected in 10 mL S-monovette and suppplemented with 2 mL of ACD pH 4.5 and then the sample was centrifuged for 20 min at 150 g at room temperature. Platelet-rich-plasma (PRP) was collected in new 15 ml tubes and supplemented with 1/10 ACD, 2 μL of apyrase/mL (0.02 U mL−1; A6410, Sigma-Aldrich) and 5 μL PGI_2_/µL (0.1 μg mL−1; P6188, Sigma-Aldrich). Platelets were pelleted by centrifugation for 10 min at 500 x *g*, washed with Tyrode’s buffer (N-2-hydroxyethyl-piperazine-N02-ethanesulphonic acid; 134 mM NaCl, 0.34 mM NaH_2_PO_4_, 2.9 mM KCl, 12 mM NaHCO_3_, 5 mM HEPES, 5 mM glucose, 0.35% BSA, pH 7.4) containing 2 µL apyrase/mL and 5 µL PGI_2_/mL and finally resuspended at a concentration of 5x10^6^/µL in human platelet poor plasma (PPP). Platelet concentrates (200 μL) were injected into the retro-orbital plexus of age- and sex-matched NOD/SCID mice (NOD.Cg-Pkrdcscid/J; Charles River, Wilmington, Massachussets, USA). Control IgG (100 µg), Emf1 IgG (10 µg) and JAQ1 IgG (100 µg) were injected i.v. in the retro-orbital venus plexus. 3 hours after antibody injection mice were bled, PRP was generated as described above and incubated with the anti-GPVI antibody Emf2^FITC^ and the anti-GPIIb antibody G8-Fab^AF546^ (unpublished). The samples were diluted in PBS and measured in FACs as described above.

Supplemental figures

**Figure S1**

**
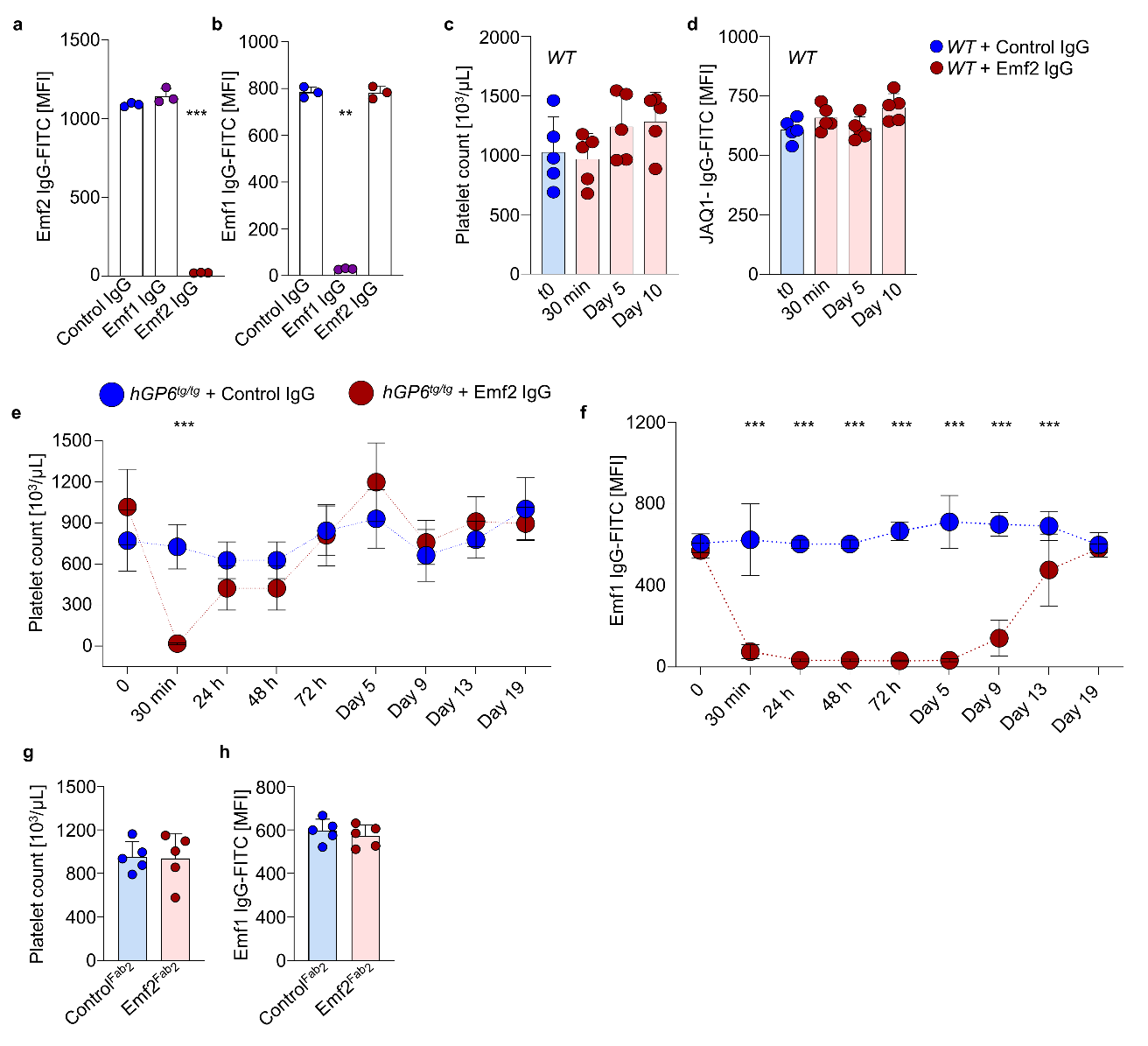
**

**Supplemental Figure 1: hGPVI downregulation is not epitope- or antibody-specific. (a-b)** Diluted blood from *hGP6^tg/tg^* mice was first incubated with either Emf1, Emf2 or control IgG, followed by staining with Emf2^FITC^ (**a**) or Emf1^FITC^ (**b**). (**c-d**) *WT* mice were treated with Emf2 (4 mg/kg b.w.) and platelet count (**c**) was determined using an automated cell counter while GPVI exposure (**d**) was measured by flow-cytometry using JAQ1^FITC^. (**e-f**) *hGP6^tg/tg^* mice (n=5) were treated with 4 mg/kg b.w. Emf2 or control IgG; platelet count (**e**) was determined using an automated cell counter while GPVI exposure (**f**) was measured by flow cytometry using Emf1^FITC^. (**g-h**) *hGP6^tg/tg^* mice were treated with 4 mg/kg Emf2^Fab2^; 1 hour after injection platelet count (**g**) was determined using an automated cell counter and GPVI surface expression (**h**) was measured by flow-cytometry using Emf1^FITC^. Data were analyzed using Two-Way ANOVA followed by Bonferroni's multiple comparison test and are expressed as mean ± SD; significance is expressed as ***p < 0.001 vs. indicated groups.

**Figure S2**


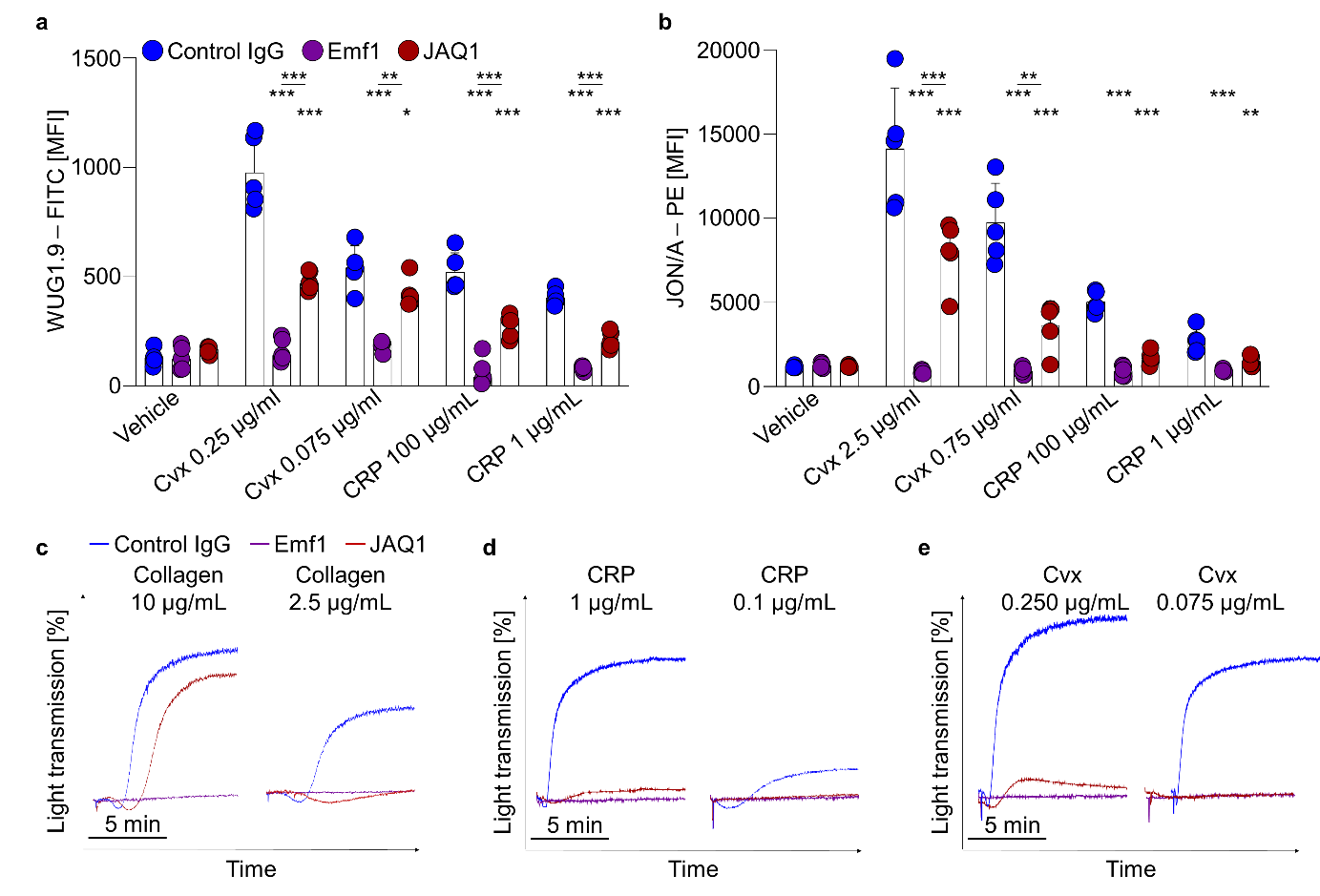


**Supplemental Figure 2: Ex vivo activation of hGPVI^KO-like^ and hGPVI^LO^ platelets.** (**a-b**) Degranulation (-WUG1.9^FITC^) (**a**) and activation of platelet αIIbβ3 integrin (JON/A^PE^) (**b**) was determined by flow cytometry upon stimulation with the indicated agonists. (**c-e**) Aggregation responses of washed platelets to high and low doses of collagen (**c**), CRP (**d**) and convulxin (**e**) in light-transmission aggregometry over 10 min (n = 4). Data were analyzed using Two-Way ANOVA followed by Bonferroni's multiple comparison test and are expressed as mean ± SD; significance is expressed as *p < 0.05, **p < 0.01, ***p < 0.001 vs. indicated groups.

**Figure S3**

**
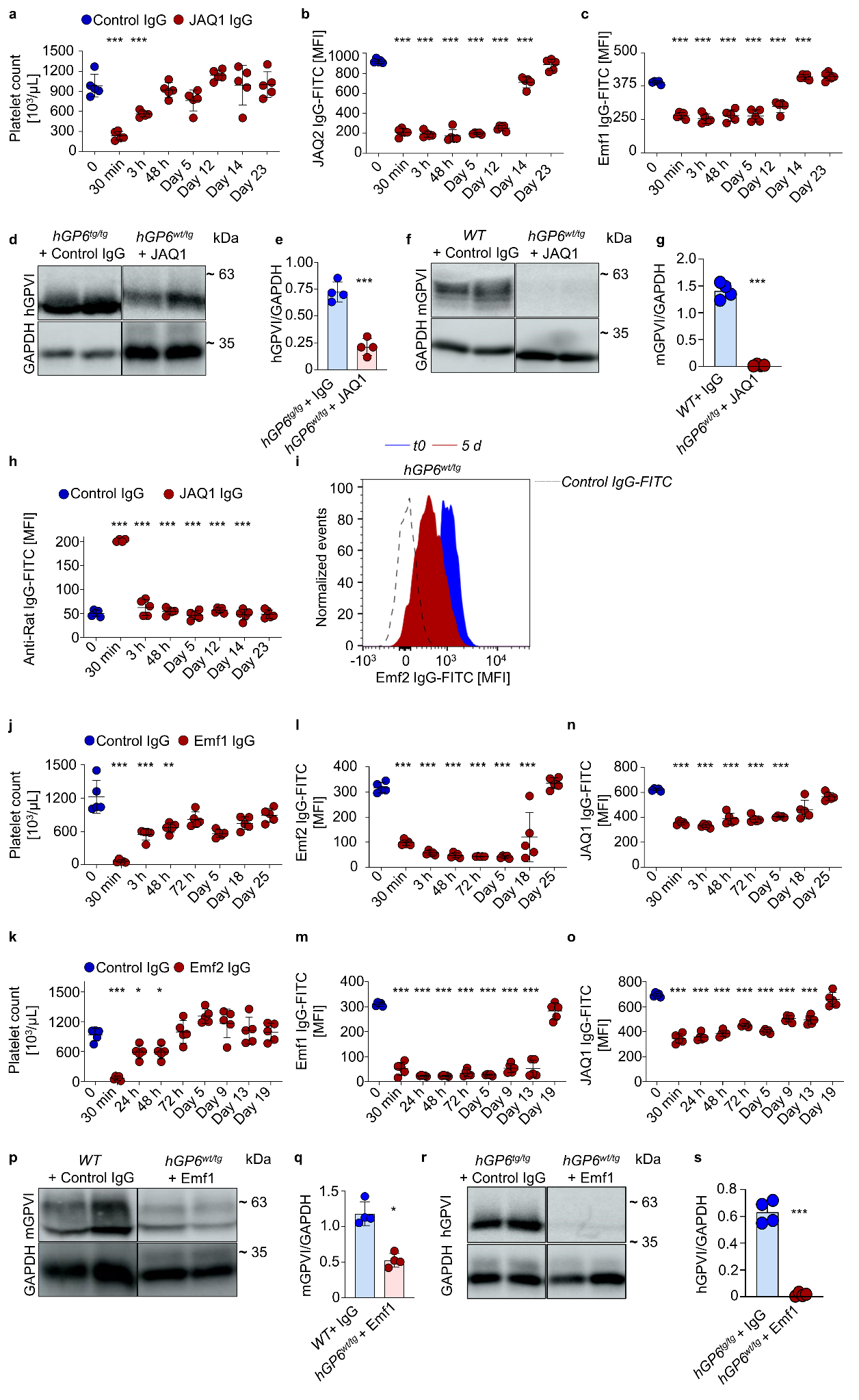
**

**Supplemental Figure 3: The generation of hGPVI^LO^ platelets is independent of receptor density.** (**a-c**) *hGP6^wt/tg^* mice (n=5) were treated with 4 mg/kg b.w. JAQ1 IgG or control IgG. Platelet count (**a**) was determined using an automated cell counter, overall GPVI exposure was measured in flow-cytometry using JAQ2^FITC^ (**b**), hGPVI exposure was measured using Emf1^FITC^ (**c**). (**d-g**) Quantitative Western blot analysis of hGPVI (**d-e**) and mGPVI (**f-g**) in platelet lysate 5 d post-injection compared to GAPDH loading control. (**h**) Receptor opsonization was measured using anti-rat-IgG^FITC^ in flow cytometry. (**i**) Flow cytometric population analysis of hGPVI receptor abundance on platelets ex vivo at t=0 or 5 d after JAQ1 IgG injection. (**j-o**) *hGP6^wt/tg^* mice (n=5) were treated with 4 mg/kg bw Emf1 (**j-l, n**), Emf2 (**k, m-o**) or control IgG. Platelet count (**j-k**) was determined using an automated cell counter, hGPVI exposure was measured in flow cytometry using Emf1 or Emf2^FITC^ (**l-m**) while overall GPVI was measured by JAQ1 IgG^FITC^ (**n-o**). (**p-s**) Quantitative Western blot analysis of mGPVI (**p-q**) or hGPVI (**r-s**) in platelet lysates 5 d post-injection compared to GAPDH loading control. Data were analyzed using the Kruskal-Wallis test (a-c, h, j-o) or the Mann-Whitney U test (d, f, q, s) and are expressed as mean ± SD; significance is expressed as *p < 0.05 **p < 0.01, ***p < 0.001 vs. indicated groups.

**Figure S4**

**
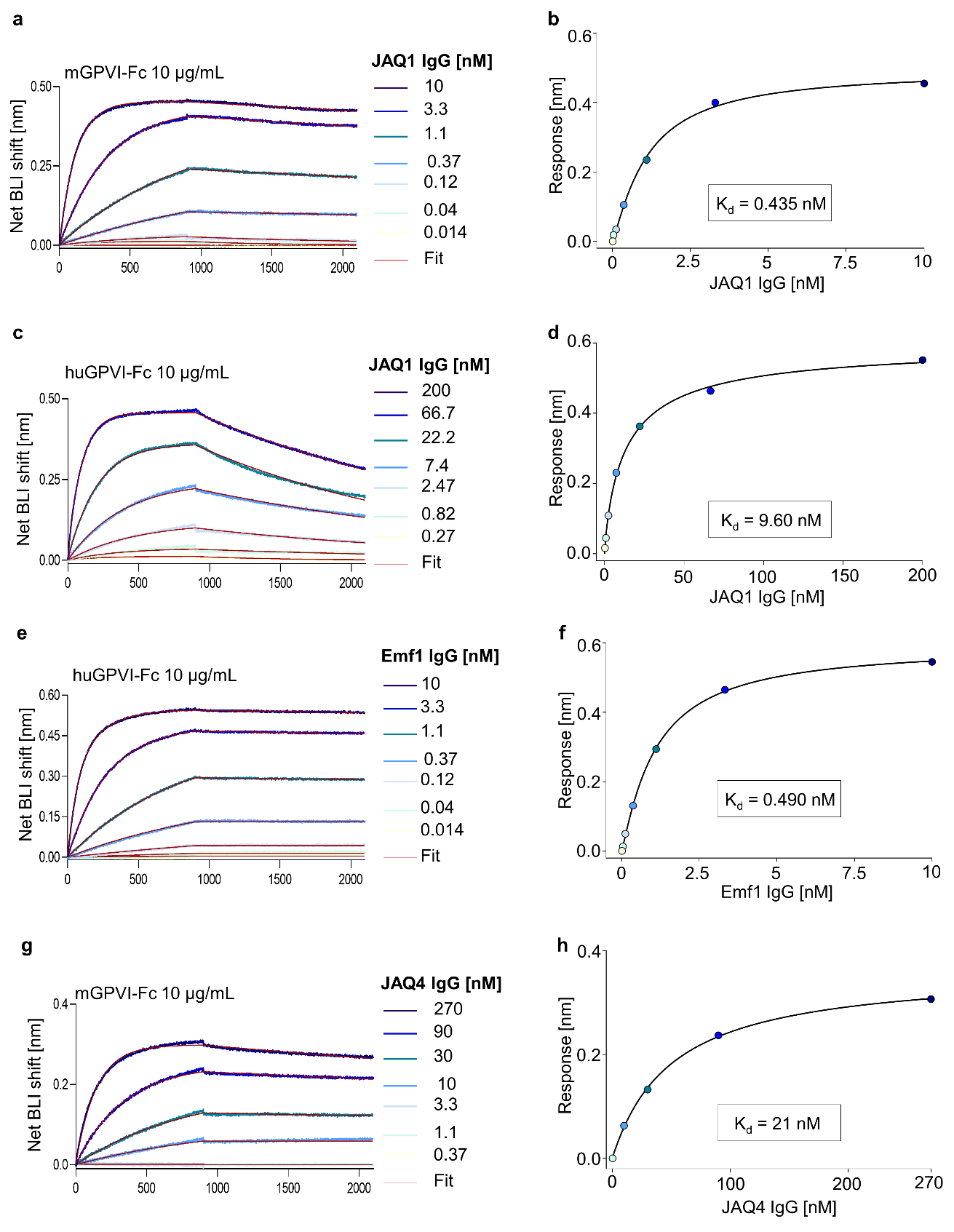
**

**Supplemental Figure 4: Affinity of the anti-GPVI antibodies used in this study.** (**a, c, e, g**) BLI sensorgrams of the association (0-900s) and dissociation (900-2100 s) showing real-time binding of the indicated mAb at various concentrations (0.014–10 nM) to immobilized mGPVI-Fc or hGPVI-Fc (10 μg/mL). Sensorgrams are overlaid with global fits to a 1:1 binding model (red lines). (**b, d, f, h**) Equilibrium binding analysis plotting the response (nm) respect to each mAb concentration. The calculated dissociation constant (Kd) is indicated.

**Figure S5**


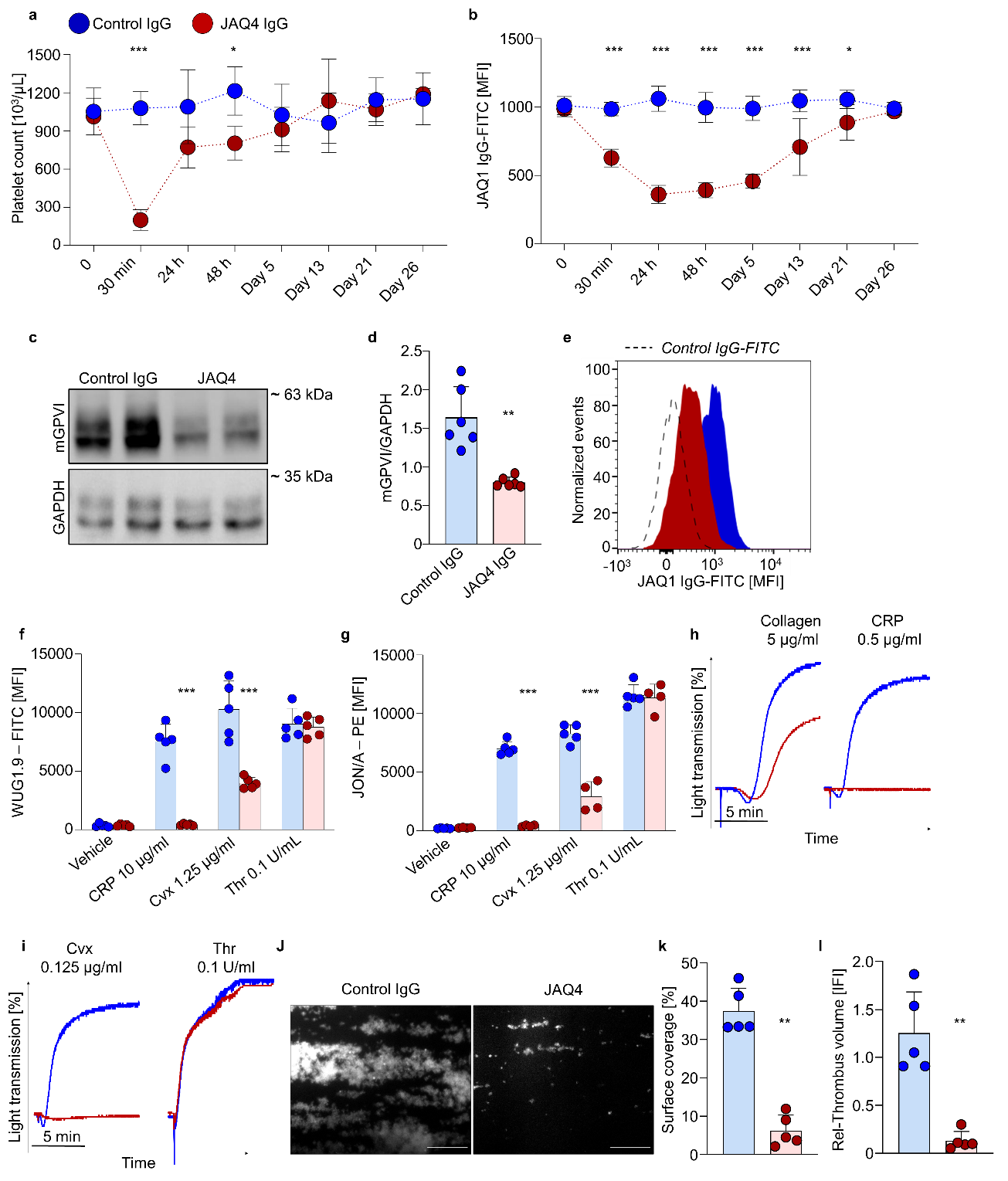


**Supplemental Figure 5: Low-affinity anti-mGPVI JAQ4 IgG induces a GPVI^LO^ phenotype. (a-b)** *WT* animals (n=5) were treated with 4 mg/kg b.w. JAQ4 IgG. Platelet count was determined using an automated cell counter (**a**), while GPVI receptor levels were measured using JAQ1^FITC^ (**b**). **(c-d)** Quantitative Western blot analysis of mGPVI in platelet lysates from *WT* animals treated with JAQ4 IgG or control IgG at 5 days post-injection compared to GAPDH loading control. **(e)** Flow cytometric-population analysis of GPVI receptor abundance on platelets ex vivo from *WT* mice 5 d after injection; dashed lines represent control IgG^FITC^. The analysis was performed using FlowJo. (**f-g**) Degranulation (Wug.E9^FITC^) (F) and activation of platelet αIIbβ3 integrin (JON/A^PE^) (G) was determined by flow cytometry upon stimulation with the indicated agonists. (**h-i**) Aggregation responses of washed platelets to the indicated agonists in light-transmission aggregometry over 10 min (n = 4); Thr = Thrombin. (**j-l**) Platelet adhesion (**k**) and aggregate formation (**l**) on Horm collagen (200 µg/mL) under flow (1000 s⁻¹) were analyzed using heparinized blood from young (8 weeks, 2 male and 3 female) *WT* mice 5 d post-injection. Representative fluorescence images of adhesion are shown (**j**, scale bar = 50 µm). Data were analyzed using Two-Way ANOVA followed by Bonferroni's multiple comparison test (a-b), the Mann-Whitney test (d,k-l) and or the Kruskal-Wallis test (f-g) and are expressed as mean ± SD; significance is expressed as **p < 0.01, ***p < 0.001 vs. indicated groups.

**Figure S6**


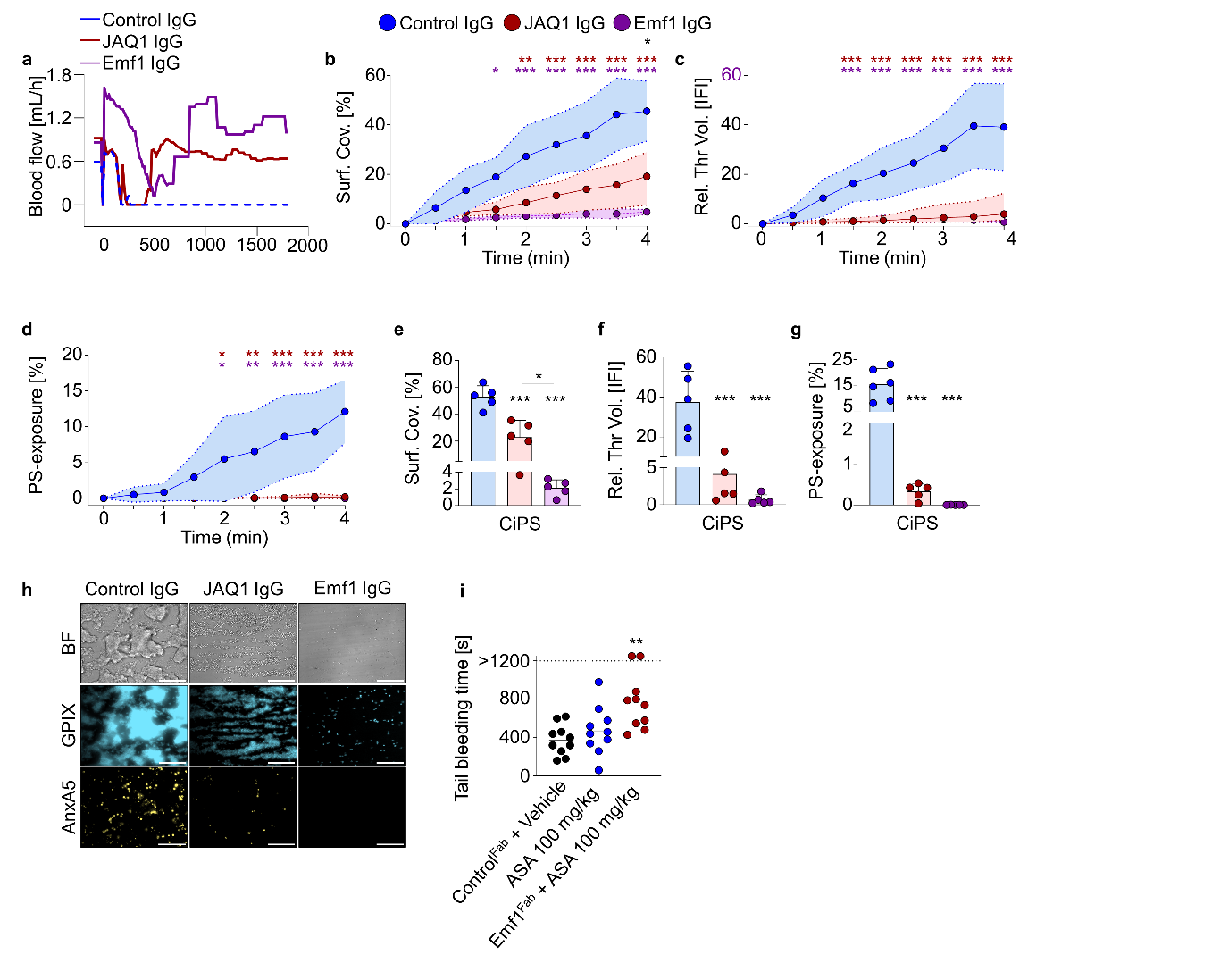


**Supplemental Figure 6: Retained GPVI adhesive function is critical to reduce bleeding tendency. (a)** Representative blood flow traces are shown from control IgG, Emf1 or JAQ1-treated young *hGP6^tg/tg^* mice. **(b-g)** Assessment of platelet adhesion (**b,e**), aggregate formation (**c,f**) and (phosphatidylserine)PS-exposure (**d,g**) over time on Horm collagen (200 µg/mL) under flow (1700 s^-1^) in heparinized blood from adult (23-27 weeks, 3 male and 2 female) *hGP6^tg/tg^* mice before (**b-d**) and after (**e-g**) perfusion of Ca^2+^ over the surface; (**h**) Representative images are shown; scale bar: 50 µm; CiPS = Calcium-induced Procoagulant State; AnxA5 = Annexin A5. **(i)** *hGP6^tg/tg^* mice were treated with either 4 mg/kg b.w. Emf1^Fab^ or control Fab, in combination with ASA 100 mg/kg or vehicle solution. Hemostasis was assessed using the tail bleeding time assay with each circle representing one mouse. Data were analyzed using the Kruskal-Wallis test as well as the Fisher´s exact test. Significance is expressed as **p < 0.01 vs individual groups.

**Figure S7**


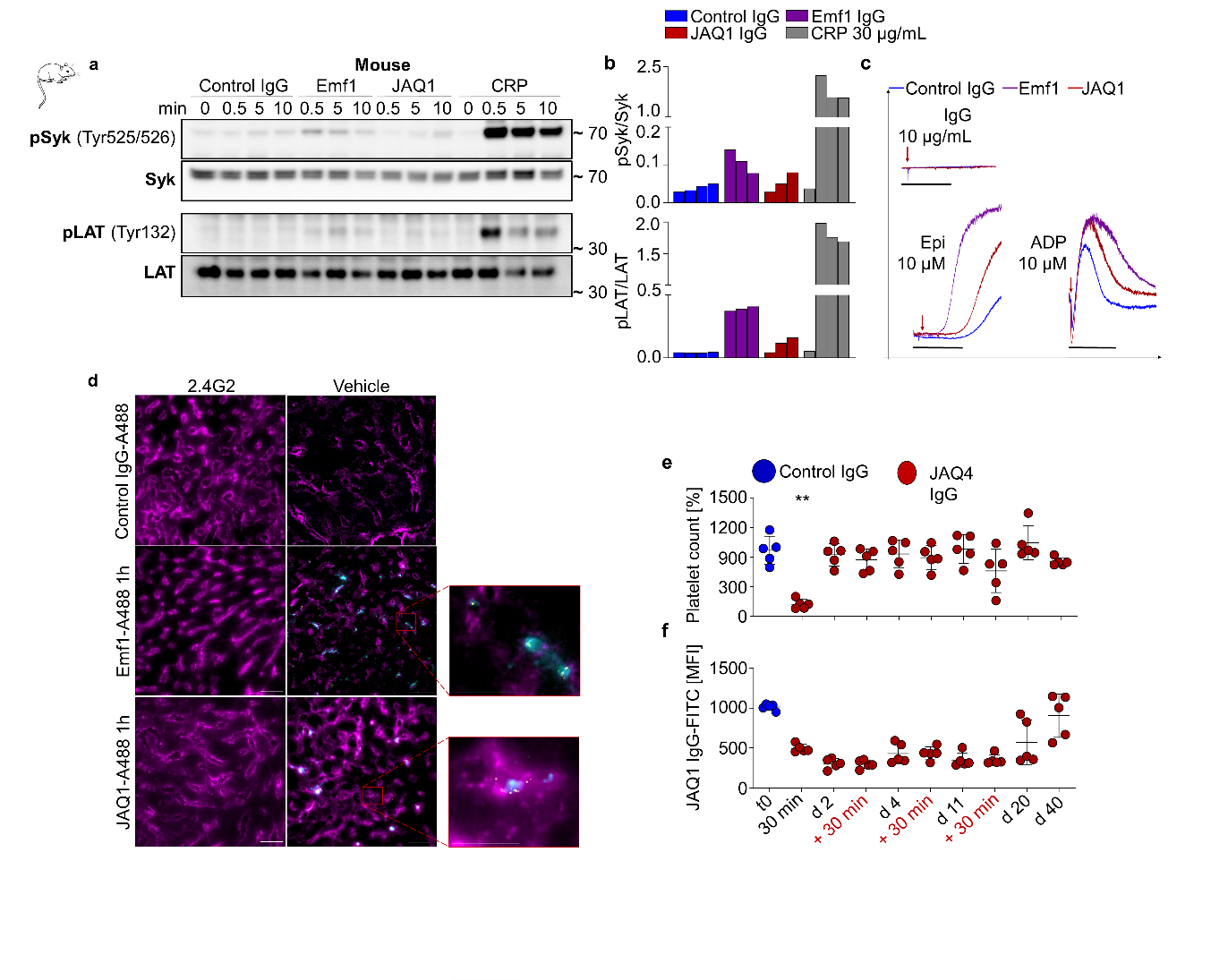


**Supplemental Figure 7: Repeated injections of JAQ4 IgG prolongs the GPVI^LO^ phenotype in *WT* mice.** (**a-b**) Western blot analyses of Syk and LAT phosphorylation in washed murine (*hGP6^tg/tg^*) platelets stimulated with Emf1, JAQ1, control IgG (20 µg/mL) or with CRP (30 µg/mL) as control. Bands (**a**) and relative quantification (**b**) are shown. (**c**) Aggregation traces (20 min) in response to the indicated IgGs, low-dose epinephrine (10 µM) or ADP (5 µM). (**d**) *hGP6^tg/tg^* mice (n = 5) were pretreated with 2.4G2 (4 mg/kg b.w.) to block FcγRIIB, followed by administration of Emf1, Emf2, JAQ1, or control IgG (4 mg/kg b.w) **(d)** Representative immunofluorescence staining of liver cryosections (7 µm) from *hGP6^tg/tg^* mice treated as indicated. Platelets were stained with anti-GPIX^AF546^ (cyan) and anti-GPVI^AF488^ (yellow); blood vessels were visualized using anti-CD31^AF647^ (purple), scale bar:50 µm; zoom in: 25µm.

(**e-f**) *WT* mice were treated with repeated injections of 4 mg/kg b.w. JAQ4 IgG. Platelet count was determined using an automated cell counter (**e**), while GPVI surface expression was measured by flow cytometry using JAQ1^FITC^ (**f**). Measurements 30 min after a new injection of the respective antibodies are labelled in red. Data were analyzed using the Friedmann Test and are expressed as mean ± SD.

**Table S1**

**Supplemental Table 1**: Detail overview of the anti-GPVI antibodies used in the article

| Clone | Host | Type | Antigen | Affinity | Source |
| --- | --- | --- | --- | --- | --- |
| JAQ1 | Rat | IgG2a κ | Mouse/Human | Mouse: 0.435 nM Human: 9.60nM | In-house |
| JAQ2 | Rat | IgG2a κ | Mouse | not determined | In-house |
| JAQ4 | Rat | IgG2a κ | Mouse | 21 nM | In-house |
| Emf1 | Mouse | IgG1 κ | Human | 0.490 nM | Emfret Analytics |
| Emf1^Fab^ | Mouse | IgG1 κ | Human | - | Emfret Analytics |
| Emf2 | Mouse | IgG1 κ | Human | not determined | Emfret Analytics |

**Supplementary References**

1 Navarro, S., Stegner, D., Nieswandt, B., Heemskerk, J. W. M. & Kuijpers, M. J. E. Temporal Roles of Platelet and Coagulation Pathways in Collagen- and Tissue Factor-Induced Thrombus Formation. *Int J Mol Sci* **23** (2021). <https://doi.org/10.3390/ijms23010358>

2 Nieswandt, B. *et al.* Expression and function of the mouse collagen receptor glycoprotein VI is strictly dependent on its association with the FcRgamma chain. *J Biol Chem* **275**, 23998-24002 (2000). <https://doi.org/10.1074/jbc.M003803200>

3 Schulte, V. *et al.* Targeting of the collagen-binding site on glycoprotein VI is not essential for in vivo depletion of the receptor. *Blood* **101**, 3948-3952 (2003). <https://doi.org/10.1182/blood-2002-10-3242>

4 Navarro, S. *et al.* Targeting of a conserved epitope in mouse and Human GPVI differently affects receptor function. *Int J Mol Sci* **23**, 8610 (2022). <https://doi.org/10.3390/ijms23158610>

5 Bergmeier, W. *et al.* Flow cytometric detection of activated mouse integrin alphaIIbbeta3 with a novel monoclonal antibody. *Cytometry* **48**, 80-86 (2002). <https://doi.org/10.1002/cyto.10114>

6 Stegner, D. *et al.* Thrombopoiesis is spatially regulated by the bone marrow vasculature. *Nat. Commun.* **8**, 127 (2017). <https://doi.org/10.1038/s41467-017-00201-7>

7 Schindelin, J. *et al.* Fiji: an open-source platform for biological-image analysis. *Nat. Methods* **9**, 666-670 (2012).
